# Constitutively active CaMKII Drives B lineage acute lymphoblastic leukemia/lymphoma in tp53 mutant zebrafish

**DOI:** 10.1371/journal.pgen.1011102

**Published:** 2023-12-20

**Authors:** Sarah C. Rothschild, Guanhua Lai, Robert M. Tombes, Wilson K. Clements

**Affiliations:** 1 Life Sciences, Virginia Commonwealth University, Richmond, Virginia, United States of America; 2 Pathology, Virginia Commonwealth University, Richmond, Virginia, United States of America; 3 Experimental Hematology, St. Jude Children’s Research Hospital, Memphis, Tennessee, United States of America; Brigham and Women’s Hospital, UNITED STATES

## Abstract

Acute lymphoblastic leukemia/lymphoma (ALL) is the most common pediatric cancer and is a malignancy of T or B lineage lymphoblasts. Dysregulation of intracellular Ca^2+^ levels has been observed in patients with ALL, leading to improper activation of downstream signaling. Here we describe a new zebrafish model of B ALL, generated by expressing human constitutively active CaMKII (CA-CaMKII) in *tp53* mutant lymphocytes. In this model, B cell hyperplasia in the kidney marrow and spleen progresses to overt leukemia/lymphoma, with only 29% of zebrafish surviving the first year of life. Leukemic fish have reduced productive genomic VDJ recombination in addition to reduced expression and improper splicing of *ikaros1*, a gene often deleted or mutated in patients with B ALL. Inhibiting CaMKII in human pre-B ALL cells induced cell death, further supporting a role for CaMKII in leukemogenesis. This research provides novel insight into the role of Ca^2+^-directed signaling in lymphoid malignancy and will be useful in understanding disease development and progression.

## Author summary

Acute lymphoblastic leukemia/lymphoma (ALL) is the most common pediatric cancer, representing approximately 3,000 new cases annually in patients under the age of 20 in the United States. Although survival rates for ALL are approximately 92%, there still remains a subset of cases that have poor outcomes that require novel model systems to understand disease etiology and identify novel targets for drug discovery. Our research identified calcium/calmodulin-dependent protein kinase, CaMKII, as a critical mediator of B cell ALL maturation. We found that zebrafish expressing activated CaMKII on a tp53 mutant background developed B cell ALL, with cancer cells visible in the marrow, spleen, and blood. These fish incorrectly expressed a key gene, *ikaros1*, which is often mutated or deleted in patients with B cell ALL. We also determined that treating human B ALL cells in culture with a drug targeting CaMKII caused the malignant cells to die. These results identified CaMKII as a key gene in B ALL development and a possible target for therapeutic intervention.

## Introduction

Acute lymphoblastic leukemia/lymphoma (ALL) is the most common pediatric cancer, representing approximately 3,000 new cases annually in patients under the age of 20 in the United States [1]. ALL originates from lymphoid progenitors in the bone marrow, with approximately 85% of pediatric lymphoid malignancies diagnosed as B lineage and 15% T lineage. While existing treatments are effective in eliminating primary disease, therapy-related complications can be severe and relapse with significantly worsened prognosis is common [2]. These concerns point to the need for additional models of ALL to better understand disease etiology, progression, relapse potential, and as a platform for identification of novel therapeutic approaches.

Zebrafish are a powerful model to study leukemia/lymphoma due to conserved regulation of normal and malignant hematopoietic behavior combined with ease of experimental manipulation and receptivity to genetic, pharmacologic, and imaging approaches [3–5]. Oncogenes, fusion proteins, and mutations are common contributors to or drivers of leukemia and lymphoma in humans and zebrafish [6–10]. Dysregulation of cytosolic calcium (Ca^2+^) levels has also been associated with leukemia in mice and humans [11,12]. One potential Ca^2+^ target is the type two Ca^2+^/calmodulin-dependent protein kinase (CaMKII). CaMKII is encoded by four highly conserved genes in mammals and seven in zebrafish; amino acid identity between human and zebrafish CaMKIIs is 90–96% [13,14]. CaMKII is expressed throughout the body and regulates diverse physiological functions [13,15–20]. It is expressed in myeloid and lymphoid lineages and has roles in immune cell maturation and response [21–23]. Dysregulation of CaMKII can lead to cancer development, including leukemia and lymphoma [21,24–26]. Cell culture and animal models of acute myeloid leukemia (AML), chronic myeloid leukemia (CML), Burkitt’s lymphoma, and ALL exhibit increased expression and activation of CaMKII [21,24,27–29]. Persistent activation of CaMKII does not require a CaMKII mutation, only persistently high Ca^2+^ levels or decreased phosphatase activity, as reported [11].

In this study, we generated a transgenic zebrafish line that expresses a phosphomimetic, constitutively active mutant (T287D) of human CaMKII (CA-CaMKII) in lymphocytes. As previously observed in mice [22,23], CA-CaMKII on its own did not induce malignancy in zebrafish; however, expression of CA-CaMKII in *tp53* mutant fish (M214K) led to an ALL phenotype. Molecular characterization of kidney marrow lymphoblasts indicated that transformation occurs in immature B cells with splenomegaly in a subset of animals. Furthermore, *rag2*:*EGFP-CA-CaMKII; tp53* mutant lymphoblasts had reduced expression and incorrect splicing of *ikaros1* (*ikzf1*), a gene commonly mutated or deleted in patients with B ALL [2,30,31], leading to altered B cell development. Finally, treatment of human pre-B ALL cells with a CaMKII inhibitor resulted in cell death, further supporting a role for CaMKII in human leukemogenesis. This new zebrafish model of B ALL provides insight into the role of Ca^2+^ signaling in B lineage leukemia and provides additional evidence that dysregulation of CaMKII can promote disease progression.

## Results

### Transgenic lymphoid expression of constitutively active CaMKII

**Fig 1:**
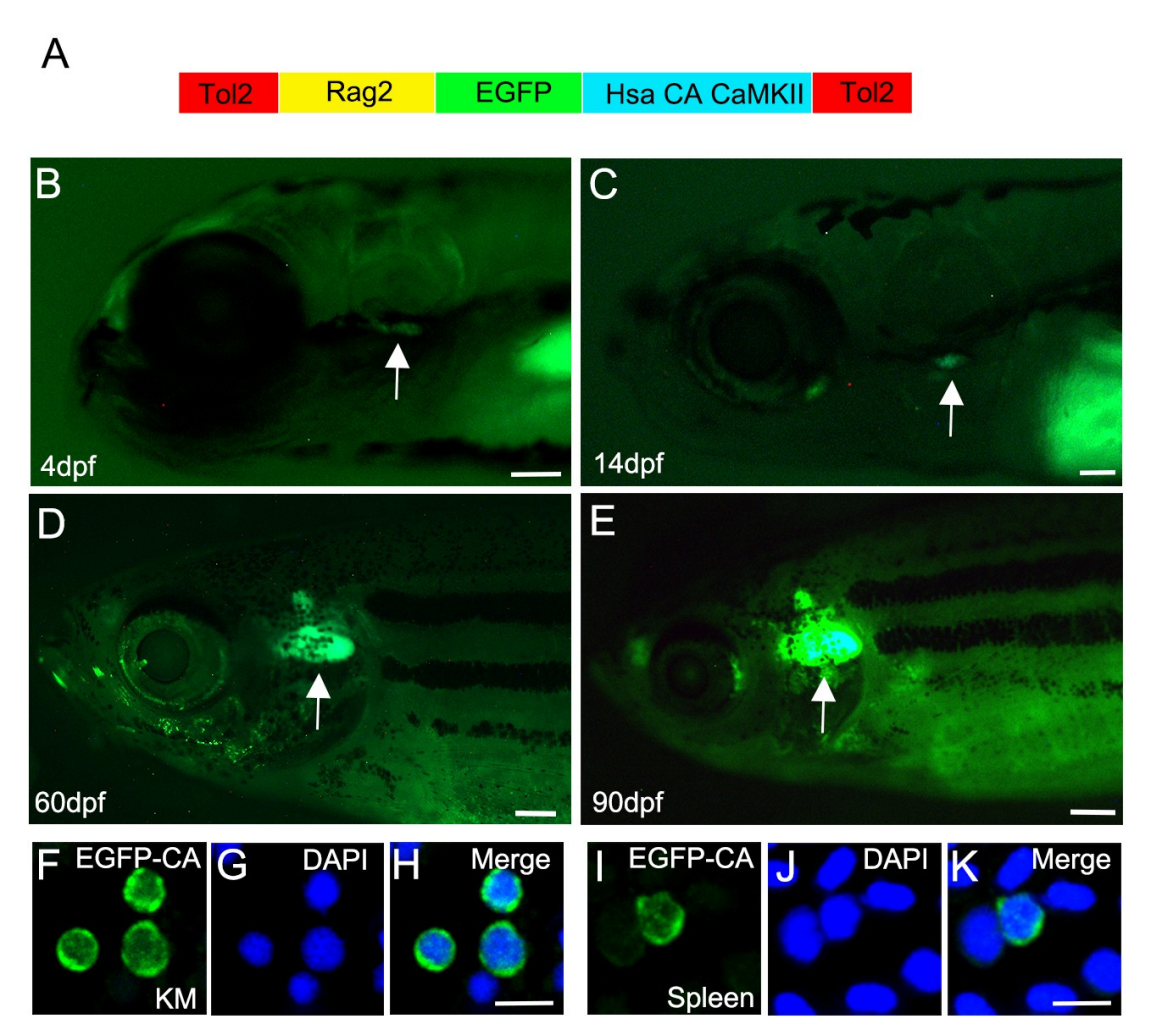
Transgenic expression of human (Hsa) constitutively active (T287D, CA) EGFP-CA-CaMKII in lymphocytes of zebrafish. **(A)** Diagram of the *rag2*:*EGFP-CA-CaMKII* construct. (B, C, D, E arrows) Stable *rag2*:*EGFP-CA-CaMKII* fish express EGFP in the thymus at 4 dpf, 14 dpf, 60 dpf, and 90 dpf. (F-K) *Rag2*:*EGFP-CA-CaMKII* is expressed in both the cytosol and nucleus of (F-H) kidney marrow (KM) and (I-K) spleen lymphocytes counterstained with DAPI at six-months of age. Scale bars: 100 μm in B and C, 400 μm in D and E, and 10 μm in H and K.

A stable transgenic zebrafish line carrying a previously characterized, constitutively active mutant (T287D) of human CaMKII (CA-CaMKII) fused with EGFP at the N-terminus [13,16,32–34] was expressed in the lymphoid lineage using the zebrafish *rag2* promoter (*rag2*:*EGFP-CA-CaMKII*) (Fig 1A) [4,7,8,35,36]. Ectopic CA-CaMKII has at least ten-fold more Ca^2+^-independent activity than wild type CaMKII [33], like that observed in leukemia/lymphoma patients [21]. Stable transgenic *rag2*:*EGFP-CA-CaMKII* animals exhibited EGFP fluorescence in the thymus starting at approximately 4 days post fertilization (dpf), and persisting through at least three months (mpf) of age (Fig 1B–1E). The observed EGFP^+^ thymus expression in *rag2*:*EGFP-CA-CaMKII* fish was identical to *rag2*:*GFP* transgenics during the first three months of development [8].

Outside the thymus, *rag2*^+^ lymphocytes are found in the kidney marrow, the site of definitive hematopoiesis, the spleen, gut, testes, ovary, and blood of adult zebrafish [35–37]. EGFP immunostaining of isolated kidney marrow and spleen cells revealed nuclear and cytosolic CaMKII (Fig 1F–1K). Given that the transgenic CaMKII peptide lacks a nuclear localization sequence, observed nuclear localization is likely due to hetero-oligomerization with endogenous nuclear CaMKII isoforms [32]. In support of this possibility, RT-PCR profiling of wild type kidney marrow lymphocytes revealed endogenous expression of both cytosolic (δ2-E; β1-C) and nuclear (β1-K; γ2-K) CaMKII isoforms [13,14]. Therefore, our stable line expresses CA-CaMKII in both the cytosol and nucleus of lymphocytes in the thymus, kidney marrow, and spleen, raising the possibility of a nuclear role for CaMKII.

### Rag2:EGFP-CA-CaMKII is expressed in T cells in the thymus and immature B cells in the kidney marrow

**Fig 2:**
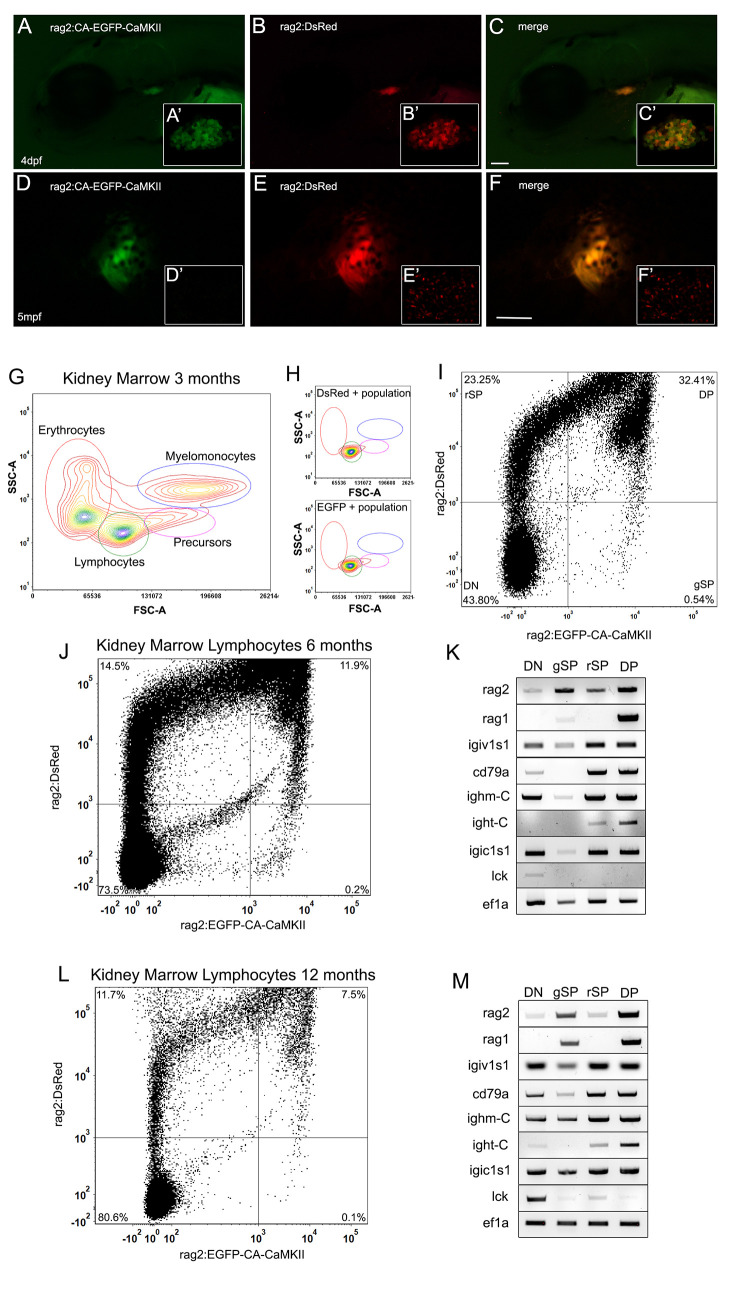
*Rag2*:*EGFP-CA-CaMKII* is expressed in thymic T cells and immature kidney marrow B lymphocytes. (A-F) *Rag2*:*EGFP-CA-CaMKII* colocalizes with *rag2*:*DsRed* in the thymus at (A-C, confocal images A’-C’) 4 dpf and (D-F) 5 mpf but does not colocalize in the skin at (skin magnified D’-F’) 5 mpf fish. (G) FACS analysis of kidney marrow cells from *rag2*:*EGFP-CA-CaMKII; rag2*:*DsRed* fish at 3 mpf. Gated populations: erythrocytes (red gate), lymphocytes (green gate), precursors (pink gate), and myelomonocytes (blue gate). (H) The EGFP and DsRed positive population for each sample is predominantly found in the lymphocyte gate, as expected. (I) Lymphocytes were further defined as double negative (DN), single positive EGFP (gSP) single positive DsRed (rSP), and double positive (DP). *Rag2*:*EGFP-CA-CaMKII; rag2*:*DsRed* kidney marrow lymphocyte cells were sorted at (J,K) 6 mpf and (L,M) 12 mpf then analyzed for B cell (*rag2*, *rag1*, *igiv1s1*, *cd79a*, *ighm-C*, *ight-C*, *igic1s1*) and T cell (*lck*), marker gene expression using RT-PCR (K,M). *Ef1a* served as a control. Scale bars: 100 μm in C and 500 μm in F.

To further confirm the tissue-specificity of our transgenic line, *rag2*:*EGFP-CA-CaMKII* fish were crossed with *rag2*:*DsRed* fish, which shows DsRed expression in T and B cells [38,39]. At 4dpf, thymocytes co-expressed DsRed and EGFP-CA-CaMKII (Fig 2A–2C and 2A’–2C’); thymic expression persisted through at least 5 mpf (Fig 2D–2F). Although DsRed^+^ cells were found on the skin at 5 mpf (Fig 2D’–2F’), these cells did not co-express EGFP-CA-CaMKII. This result was consistent with the *rag2*:*GFP* transgenic line, where GFP positive cells were not reported on the skin [4,8]. It is likely the stability of DsRed, due to the tetrameric structure, enabled the persistence of fluorescence in mature B cells, while EGFP-CA-CaMKII was degraded [40]. Taken together, this suggested that EGFP-CA-CaMKII was expressed in thymic T cells but was not expressed in mature B lymphocytes on the skin [41,42].

To evaluate the extent of transgene expression in kidney marrow, we analyzed the major hematopoietic lineages by light scatter [5] and were able to define erythrocyte, myeloid, precursor and lymphocyte populations from *rag2*:*EGFP-CA-CaMKII*; *rag2*:*DsRed* fish (Fig 2G). DsRed^+^ and EGFP^+^ populations were found almost exclusively in the lymphoid gate, as expected (Fig 2H). Further analysis of fluorophore expression revealed the presence of EGFP single positive (gSP), DsRed single positive (rSP) and EGFP; DsRed double-positive (DP), and double-negative (DN) fractions (Fig 2I), suggesting the possibility that EGFP-CA-CaMKII was expressed in a specific lymphocyte population.

To determine the significance of these fractions, we sorted and analyzed them for the expression of lymphoid markers at 6 mpf (Fig 2J and 2K) and 12 mpf (Fig 2L and 2M). We examined markers of B cell *(rag1*, *rag2*, *igiv1s1*, *ighm-C*, *ight-C*, *cd79a*, *igic1s1*) and T cell (*lck*) development. B cells display ordered gene expression indicative of maturation stage [43]. Based on expression of selected marker genes, the most immature population was the gSP (EGFP^+^) population, which expressed *rag genes* did not express or weakly expressed cd79a, and expressed low levels of immunoglobulin heavy (*ighm*, *ight*) and light chain (*igiv1s1*, *igic1s1*) genes. By the time B cells reached the DP (EGFP^+^DsRed^+^) stage, they strongly expressed *rag* genes. They then matured to the rSP (DsRed^+^) stage where *rag2* expression was reduced and *rag1* was absent. At the DN (EGFP^-^DsRed^-^) stage, no *rag1* was detectable and *rag2* was barely visible. The DN fraction also contained *lck*^*+*^ cells, which were likely T cells, although recent published data demonstrates weak *lck* expression in some B cells [7]. The generation of separate fluorescent gated populations was unexpected given both DsRed and EGFP-CA-CaMKII were expressed using the *rag2* promoter. The presence of the gSP fraction was likely due to the more rapid maturation of EGFP compared to DsRed, [40] which accounted for the small percentage of gSP cells identified in the kidney marrow (0.10–0.54%). Furthermore, the presence of rSP cells in the kidney marrow, similar to DsRed+ cells on the skin, suggested EGFP-CA-CaMKII was degraded as cells matured, while DsRed persisted due to the stability of the tetramer [40]. The differential gene expression observed at 6 and 12 mpf could be due to alterations in B cell generation and differentiation associated with aging, similar to humans [44]. Thus, the most immature B cells, due to expression of *rag1* [43], were found in the gSP and DP population, while the most mature B cells were found in the rSP and DN fractions. Therefore, we concluded that EGFP-CA-CaMKII is expressed in immature B cells.

### Lymphocyte hyperplasia in rag2:EGFP-CA-CaMKII; tp53 mutants

*Rag2*:*EGFP-CA-CaMKII* fish were phenotypically normal and displayed no significant health issues, similar to mice expressing CA-CaMKII in thymocytes [22]. Although leukemia/lymphoma patients often display increased CaMKII activity [21,24,25], constitutive activation of CaMKII alone does not appear to drive leukemic transformation, suggesting that additional genetic alterations are required.

Inactivating mutations in the tumor suppressor *TP53* occur in approximately 5–16% of ALL patients [45–49]. Although discovery mutations in TP53 often occur in conjunction with a low hypodiploid karyotype, 10–20% of relapse ALL patients have somatic mutations in TP53 without this subtype [48,50]. These TP53 alterations lead to reduced function and are linked with treatment failure [46–48,50] identifying the need for novel targets for therapeutic intervention. TP53 is activated in response to DNA damage, and is a key mediator of tumor suppression responses by pathways including ARF (CDKN2A), leading to cell cycle arrest or apoptosis. Mutations and deletions in ARF occur frequently in ALL [51], inhibiting TP53-dependent apoptosis. A zebrafish ortholog of ARF has not been identified [52], but *tp53* mutant zebrafish have been used to study interruption of the ARF-dependent signaling pathway as well as directly study *tp53* function in leukemic transformation [52]. The established zebrafish *tp53* mutant line carries a point mutation in the region encoding the DNA binding domain (M214K), leading to transcriptional inhibition of target genes. Homozygous *tp53* mutants are viable, with less than 5% of animals spontaneously developing malignancies by one year of age. Tumor incidence was estimated at 28% by 16 months, with the majority of animals developing malignant peripheral nerve sheath tumors (MPNST), but not leukemia [53]. *Rag2*:*EGFP-CA-CaMKII* fish were crossed into the *tp53* mutant background to examine their combinatorial potential to drive leukemic transformation.

**Fig 3:**
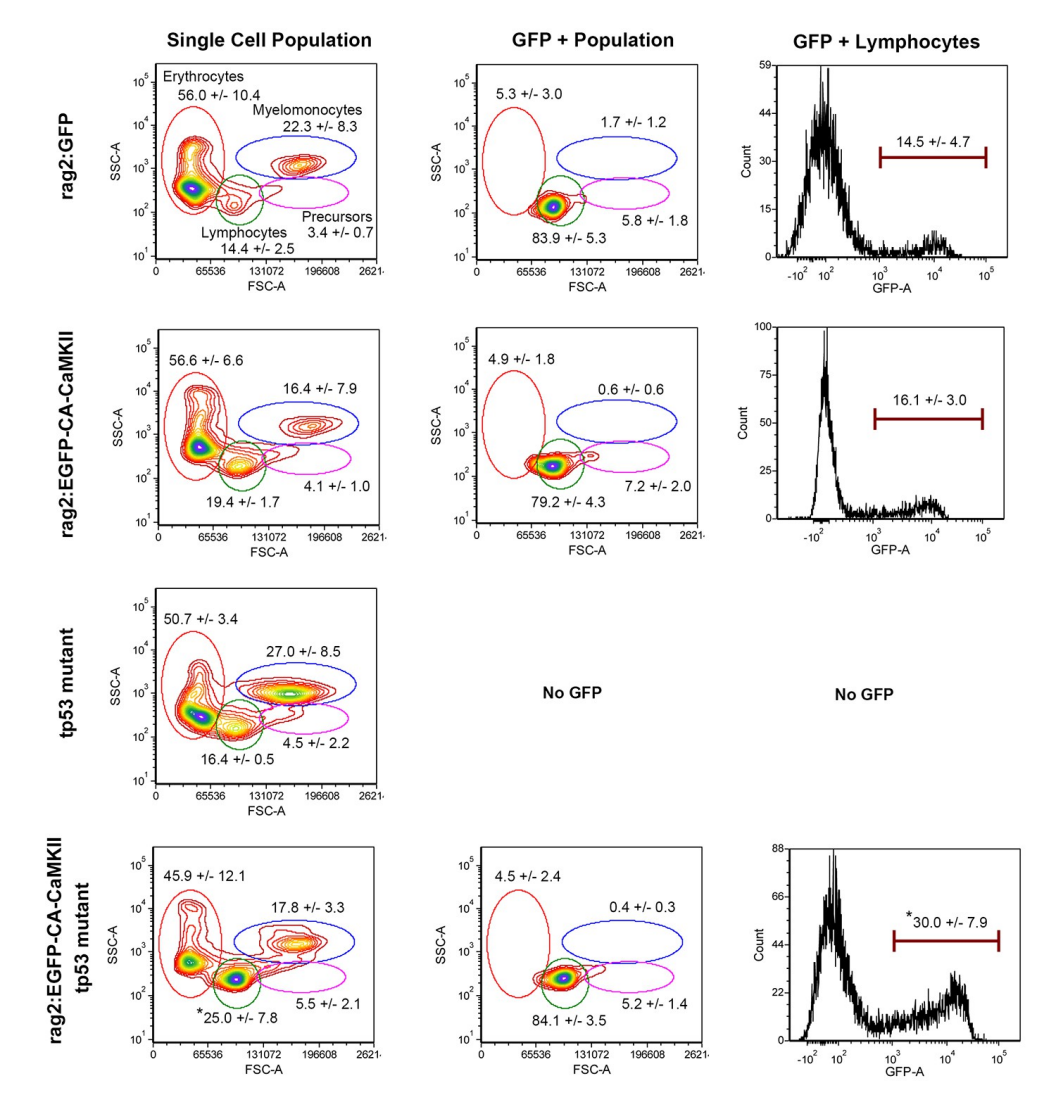
The lymphocyte population is expanded in the kidney marrow of *rag2*:*EGFP-CA-CaMKII; tp53* mutant fish. FACS analysis of the kidney of six-month old *rag2*:*GFP* (n = 4), *rag2*:*EGFP-CA-CaMKII* transgenic (n = 5), *tp53* mutant (n = 2), and *rag2*:*EGFP-CA-CaMKII; tp53* mutant fish (n = 5) without overt illness. Gated populations: erythrocytes (red), lymphocytes (green), precursors (pink), and myelomonocytes (blue). Populations of cells within each gate are described as a percentage of total live cells. The GFP positive population for each sample is predominantly found in the lymphocyte gate, as expected. The percentages of GFP positive cells in the lymphocyte gate are represented in the histograms. P values were calculated using one-way ANOVA followed by Tukey HSD. * p<0.05.

To determine if expression of *rag2*:*EGFP-CA-CaMKII* altered lymphocyte numbers in a *tp53* mutant background, flow cytometry of pooled hematopoietic cells from the kidney marrow (Fig 3) was performed on animals with and without the *EGFP-CA-CaMKII* transgene. FACS analysis revealed similar kidney marrow lymphocyte populations in *rag2*:*GFP*, *rag2*:*EGFP-CA-CaMKII* transgenic, and *tp53* mutant fish (14–19%), but elevated levels (25%) in *rag2*:*EGFP-CA-CAMKII; tp53* mutants (Fig 3). Consistent with our expectations, EGFP+ cells were primarily identified in the lymphoid gate (78–84%). The percentage of EGFP+ lymphocytes roughly doubled from 16% in *EGFP-CA-CaMKII* to 30% in *rag2*:*EGFP-CA-CaMKII; tp53* mutant kidney marrow (Fig 3).

**Fig 4:**
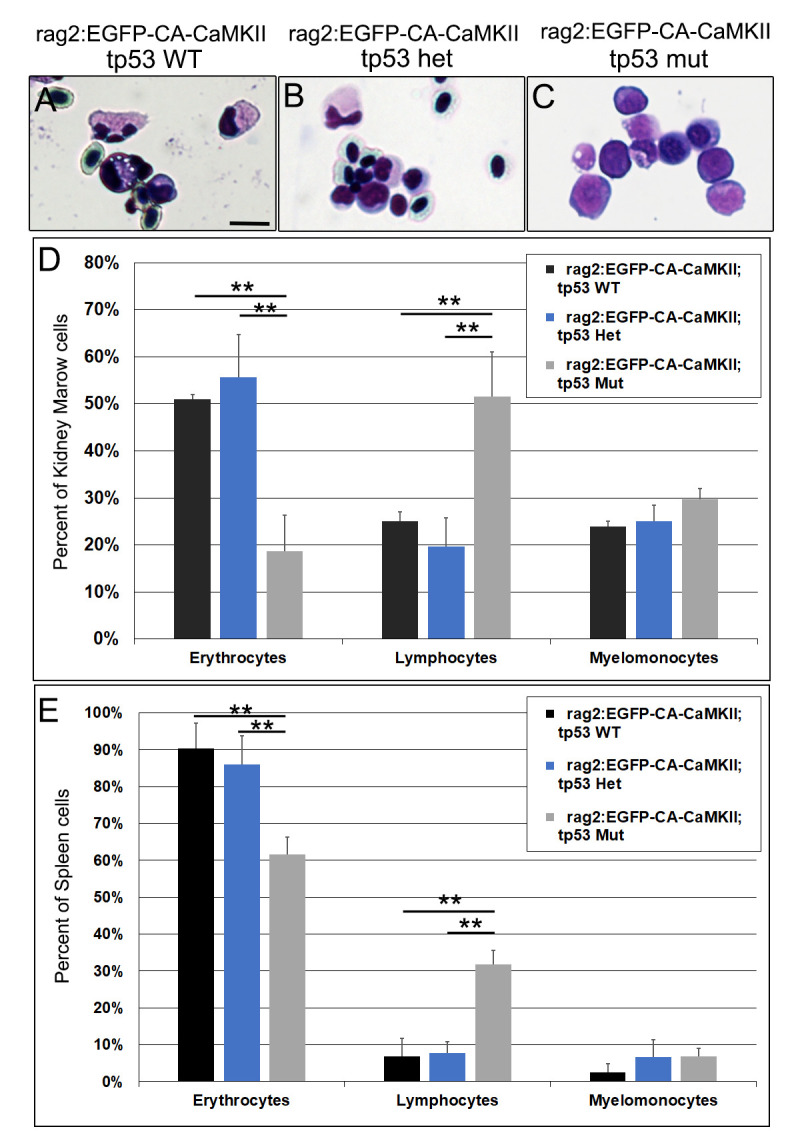
Lymphocytes are significantly expanded in the kidney marrow and spleen of *rag2*:*EGFP-CA-CaMKII; tp53 mutant* fish. Lineage distributions as determined by cell morphology in the (A-D) kidney marrow and (E) spleen of 9–12 month old fish segregated into *rag2*:*EGFP-CA-CaMKII* on a wild type (black, n = 3), *tp53* heterozygous (blue, n = 3), or *tp53* mutant background (gray, n = 4). Cell populations were identified from eight to ten images of HEMA3 stained blood smears per fish. P values were calculated using one-way ANOVA followed by Tukey HSD. ** p<0.01. Scale bar is 10 μm in A.

Leukemia in humans is diagnosed when lymphoblasts exceed 25% of the cell population in the bone marrow or peripheral blood, in accordance with the National Comprehensive Cancer Network guidelines [54]. The elevated lymphocyte counts observed in some individual zebrafish exceeded the threshold for diagnosis of leukemia (Fig 3). Since initial FACS analyses were conducted on fish prior to overt signs of illness, we prepared kidney marrow (Fig 4A–4C) and spleen smears from adult zebrafish that appeared ill, as demonstrated by lethargy and failure to eat. The proportion of erythrocytes, lymphocytes, and myelomonocytes in the kidney marrow (Fig 4D) and spleen (Fig 4E) was unchanged in fish carrying the *rag2*:*EGFP-CA-CaMKII* transgene on a wild type or *tp53* heterozygous background. However, the percentage of erythrocytes was significantly decreased and the percentage of lymphocytes more than doubled in the kidney marrow and spleen of *rag2*:*EGFP-CA-CaMKII; tp53* mutants. (Fig 4D and 4E). Thus, *rag2*:*EGFP-CA-CaMKII; tp53* mutants progress from lymphoid hyperplasia to overt malignancy.

### ALL transformation in rag2:EGFP-CA-CaMKII; tp53 mutants

**Fig 5:**
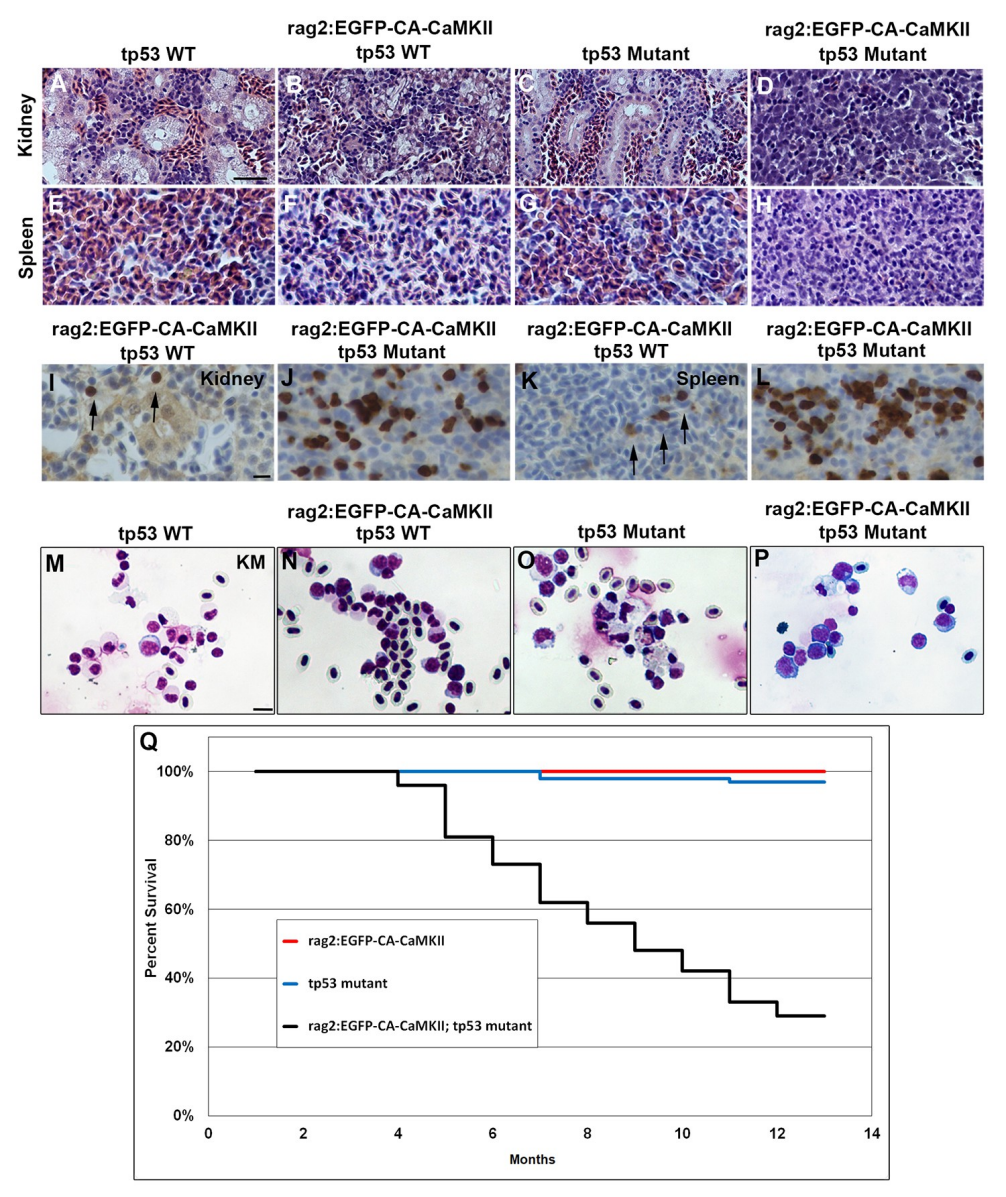
*Rag2*:*EGFP-CA-CaMKII; tp53* mutant fish develop acute lymphoblastic leukemia/lymphoma. Hematoxylin and eosin stained sections of the (A-D) head kidney and (E-H) spleen of nine month old (A, E) wild type, (B, F) *rag2*:*EGFP-CA-CaMKII* transgenic, (C, G) *tp53* mutant, and (D, H) *rag2*:*EGFP-CA-CaMKII; tp53* mutant fish. (D, H) An increase in lymphoblasts is observed in the kidney and spleen of *rag2*:*EGFP-CA-CaMKII; tp53* mutant fish. (I-L) Anti-EGFP immunohistochemistry indicates EGFP positive lymphocytes are increased in the (J) kidney marrow and (L) spleen of leukemic fish compared to (I, K) *rag2*:*EGFP-CA-CaMKII* wild type fish. Kidney marrow (KM) cytospins of (M) wild type, (N) *rag2*:*EGFP-CA-CaMKII* transgenic, (O) *tp53* mutant, and (P) *rag2*:*EGFP-CA-CaMKII; tp53* mutant were HEMA3 stained. (Q) Four cohorts of fish were assessed for survival during the first year. p<0.0001 comparing *rag2*:*EGFP-CA-CaMKII; tp53* wild type fish to *rag2*:*EGFP-CA-CaMKII*; *tp53* mutants and *tp53* mutants to *rag2*:*EGFP-CA-CaMKII*; *tp53* mutants for survival using the Log-rank (Mantel-Cox) test. A statistically significant reduction in survival of *rag2*:*EGFP-CA-CaMKII; tp53* fish was evident, p<0.01. Scale bars: 40 μm in A and 10 μm in I and Q.

To further understand this pathology, we performed histology on *rag2*:*EGFP-CA-CaMKII; tp53* animals that were lethargic and failing to thrive. Sections from wild type and *rag2*:*EGFP-CA-CaMKII* transgenic fish revealed normal pronephric tubules and hematopoietic cells (Fig 5A and 5B). *Tp53* mutant fish also maintained normal kidney morphology, but showed an increase in myelomonocytic cells (Fig 5C). However, *rag2*:*EGFP-CA-CaMKII; tp53* mutant fish lost normal kidney morphology with immature lymphoblasts identified throughout the kidney (Fig 5D). Like kidney marrow, the spleen was normal in wild type, *rag2*:*EGFP-CA-CaMKII* transgenic, and *tp53* mutant fish (Fig 5E–5G). In contrast, *rag2*:*EGFP-CA-CaMKII; tp53* mutant spleens were infiltrated with immature lymphoblasts (Fig 5H), and splenomegaly was detected in 14% of fish (S1 Fig). Notably, the thymus, gills, liver, and muscle tissue appeared normal in all samples (S2 Fig). Furthermore, circulating EGFP-CA-CaMKII lymphoblasts were increased in *rag2*:*EGFP-CA-CaMKII; tp53* mutants compared to *rag2*:*EGFP-CA-CaMKII* fish (S1 and S2 Videos). Anti-GFP immunohistochemistry confirmed increased EGFP expression in the kidney marrow (Fig 5J) and spleen (Fig 5L) of leukemic fish compared to *rag2*:*EGFP-CA-CaMKII* (Fig 5I and 5K). To further validate leukemia development in *rag2*:*EGFP-CA-CaMKII;tp53* mutant fish, kidney marrow blood smears were analyzed. While a mixture of erythrocytes, lymphocytes and myelomonocytes were observed in wild type, *rag2*:*EGFP-CA-CaMKII* transgenic, and *tp53* mutant fish (Fig 5M–5O), immature lymphoblasts, determined by high nuclear to cytoplasmic ratio, large size, and immature chromatin, comprised the majority of kidney marrow cells in *rag2*:*EGFP-CA-CaMKII; tp53* mutants (Fig 5P).

Survival rates were compiled across four cohorts of *rag2*:*EGFP-CA-CaMKII* transgenic, *tp53* mutant, and *rag2*:*EGFP-CA-CaMKII; tp53* mutant fish during the first twelve months of life (Fig 5Q). Analyses were limited to this time frame since *tp53* mutant fish begin developing spontaneous cancers at approximately 8 months of age [53]. All of the *rag2*:*EGFP-CA-CaMKII* fish (n = 235) on a wild type background survived and two out of fifty-six *tp53* mutant fish died of MPNST, consistent with previously reported rates [53]. In contrast, thirty-seven out of fifty-two *rag2*:*EGFP-CA-CaMKII; tp53* mutant fish died within the first year. Of these 37 fish, 5% (n = 2) exhibited MPNST, 14% (n = 5) showed increased immature lymphoblasts without overt leukemia/lymphoma, 35% (n = 13) died prior to analysis, and 46% (n = 17) developed ALL (Fig 5U). Overt illness occurs rapidly, where fish with leukemia/lymphoma were unable to eat and succumb to illness within four-to-six hours, resulting in 35% of fish dying prior to analysis. However, leukemia developed solely in fish that expressed lymphoid targeted *EGFP-CA-CaMKII* in *tp53* mutants, further suggesting inappropriate activation of CaMKII is a second hit leading to leukemic transformation.

### Rag2:EGFP-CA-CaMKII is expressed in kidney marrow B cells

**Fig 6:**
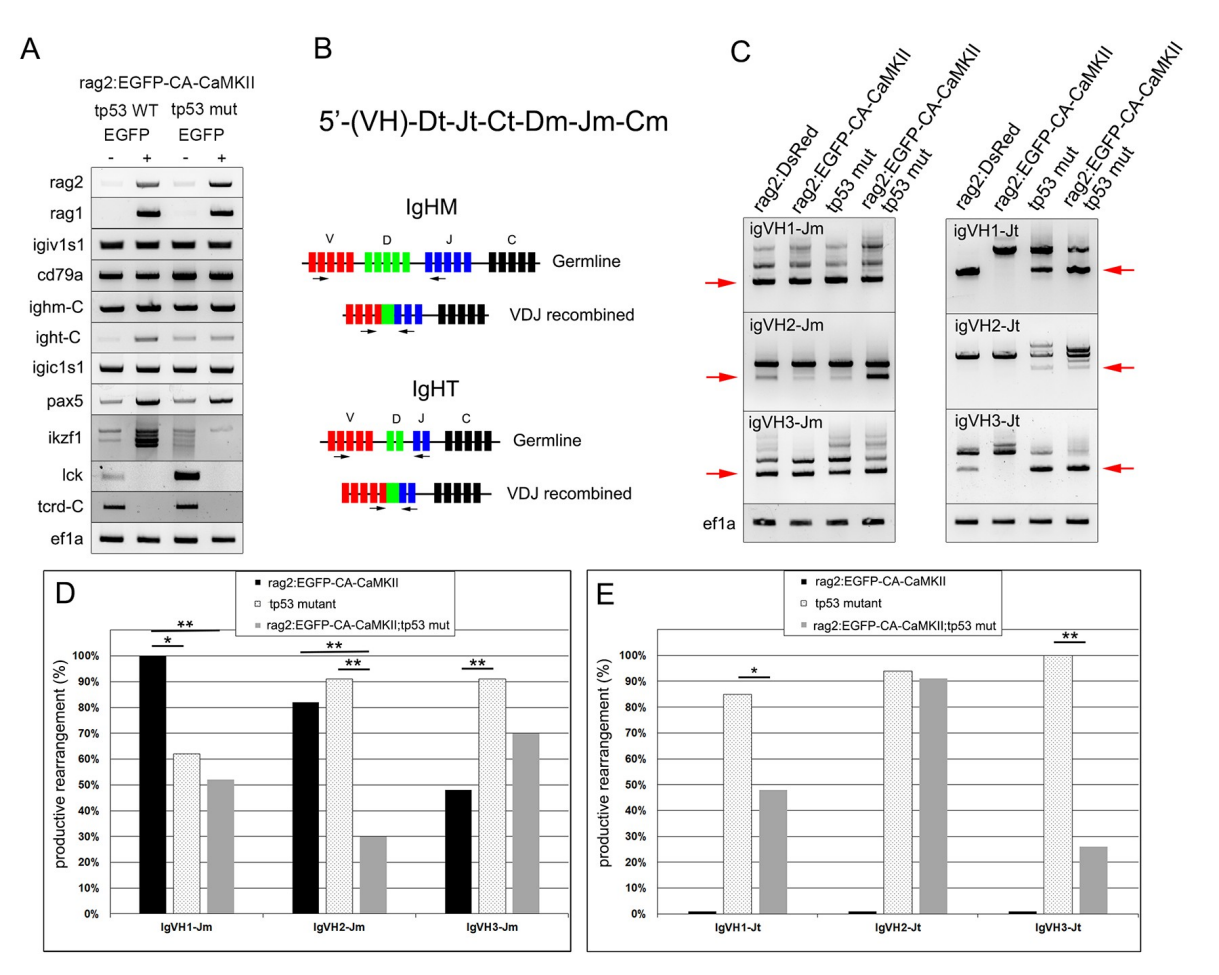
Kidney marrow blasts expressing *rag2*:*EGFP-CA-CaMKII* are B lineage and have reduced productive IgH VDJ rearrangements. EGFP positive and negative lymphoid cells from the kidney marrow were sorted from six-nine month old adult *rag2*:*EGFP-CA-CaMKII* transgenics on a wild-type or *tp53* mutant background and the expression of B (*rag2*, *igiv1s1*, *cd79a*, *ighm-C*, *igt-C*, *pax5*, *ikzf1*) and T (*lck*, *tcrd-C*) lineage markers was determined by RT-PCR. *Ef1a* served as control (A). Arrangement of the zebrafish IgH locus and schematic of IgHM and IgHT VDJ assembly (B). Genomic analysis of VDJ recombined (red arrow) IgVH1-Jm, IgVH2-Jm, IgVH3-Jm, IgVH1-Jt, IgVH2-Jt, and IgVH3-Jt in *rag2*:*DsRed*, *rag2*:*EGFP-CA-CaMKII*, *tp53* mutant, and *rag2*:*EGFP-CA-CaMKII; tp53* mutant kidney marrow lymphocytes using semi-nested PCR. *Ef1a* served as a control (C). Analysis of productive IgHM (D) and IgHT (E) rearrangements in genomic DNA *from rag2*:*EGFP-CA-CaMKII*, *tp53* mutant, and *rag2*:*EGFP-CA-CaMKII; tp53* mutant kidney marrow lymphocytes. P values were calculated using Fisher’s exact test. * p<0.05, ** p<0.01.

To assess lineage in leukemic fish, we sorted and transcriptionally profiled EGFP positive and negative lymphocyte populations from the kidney marrow of *rag2*:*EGFP-CA-CaMKII* transgenics, either on a wild type or mutant *tp53* background, for a limited set of lineage markers. EGFP^+^ populations expressed genes associated with immature lymphoid (*rag2*, *rag1*) blasts of the B (*igiv1s1*, *cd79a*, *ighm-C*, *ight-C*, *pax5*, *igic1s1*), but not T lineage (*lck*, *tcrd-C*) (Fig 6A). Interestingly, unlike other B cell markers, expression of *ikaros1* (*ikzf1*) was reduced in EGFP^+^ *rag2*:*EGFP-CA-CaMKII; tp53* mutant lymphocytes compared to EGFP^+^ *rag2*:*EGFP-CA-CaMKII; tp53* wild type lymphocytes (Fig 6A).

To further assess B cell development, kidney marrow lymphocytes were analyzed for genomic rearrangement of the variable (V), diversity (D), and joining (J) gene segments of the immunoglobulin heavy chain locus in *rag2*:*DsRed*, *rag2*:*EGFP-CA-CaMKII*, *tp53* mutant, and *rag2*:*EGFP-CA-CaMKII; tp53* mutant fish (Fig 6C). The *igh* locus is comprised of a cluster of V gene segments followed by DJ constant (C) gene segments for *igt* and then DJC gene segments for *igm* [55–57]. In order for *igm* rearrangement to occur, the intervening *igt* DJC sequence is deleted (Fig 6B) [56]. Therefore, kidney marrow lymphocytes were assayed for productive rearrangements of *igt* and *igm* from variable domains 1, 2, and 3 to (DJ)_T_ or (DJ)_M_ by RT-PCR (Fig 6B). *Igm* VDJ-rearrangement was observed in lymphocytes of all fish examined (Fig 6C), however *rag2*:*EGFP-CA-CaMKII* fish had reduced productive IgVH3-Jm rearrangement, while *tp53* mutant fish had reduced IgVH1-Jm rearrangement and *rag2*:*EGFP-CA-CaMKII; tp53* mutant fish had reduced productive IgM rearrangement from all three V domains analyzed (Fig 6D). Unlike *igm*, *igt* rearrangements were not identified in all fish (Fig 6C, red arrows), where *rag2*:*EGFP-CA-CaMKII* fish failed to undergo V to DJ rearrangement in all samples analyzed (Fig 6C). In contrast, *tp53* mutant fish displayed productive rearrangements for all samples, while *rag2*:*EGFP-CA-CaMKII; tp53* mutant fish had reduced productive rearrangements for IgVH1-Jt and IgVH3-Jt but normal IgvH2-Jt rearrangements (Fig 6E). Our findings point to two main conclusions: First, *rag2*:*EGFP-CA-CaMKII* fish rearranged *igm* loci in preference to *igt* on a wild type background. Second, productive rearrangements are reduced in both the *igt* and *igm* loci of *rag2*:*EGFP-CA-CaMKII;tp53* mutant fish.

### Ikzf1 splicing is altered in rag2:EGFP-CA-CaMKII; tp53 mutant positive B cells

**Fig 7:**
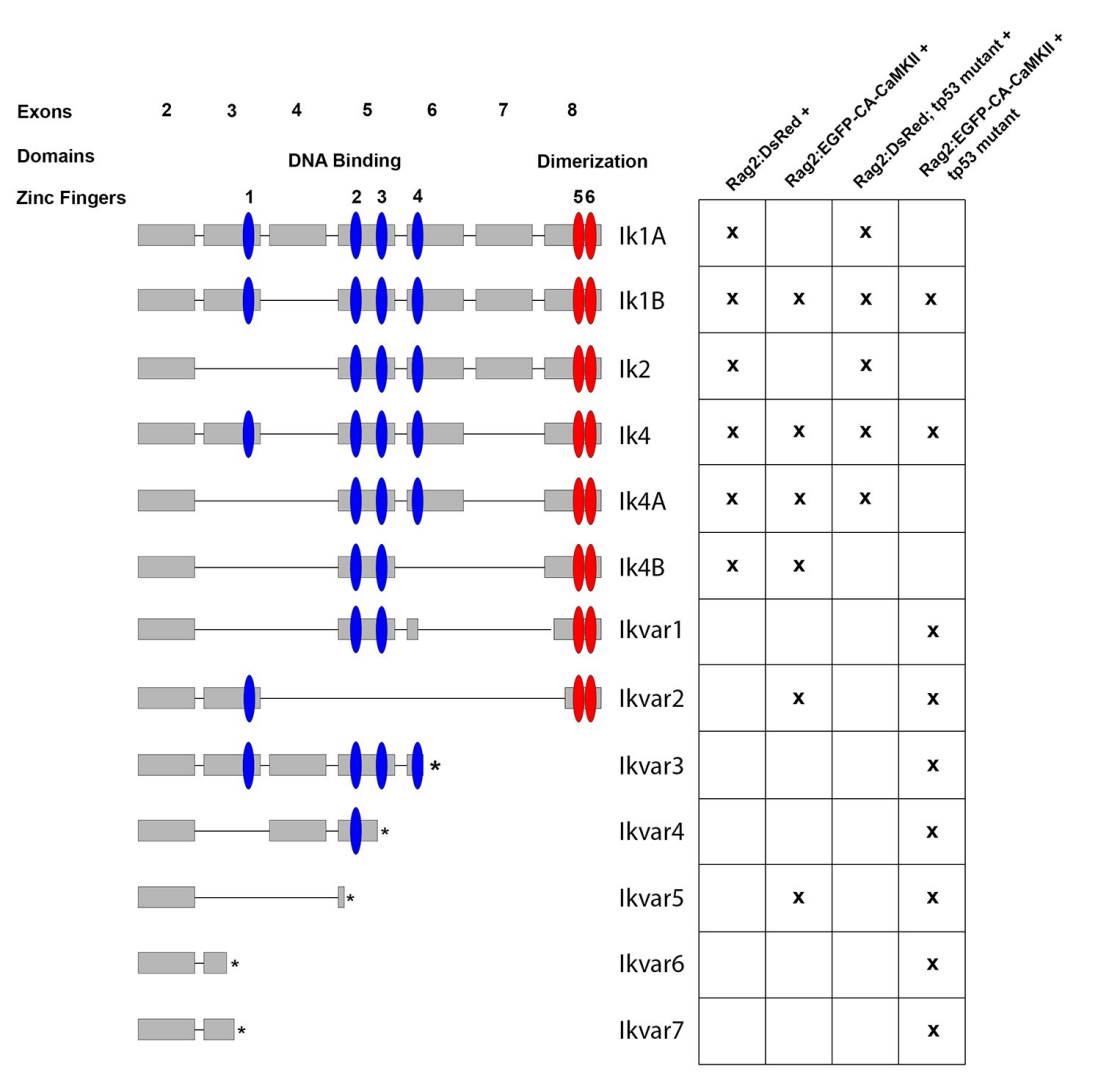
Izf1 splicing is altered in rag2:EGFP-CA-CaMKII;tp53 mutant B cells. Zebrafish *ikzf1* was PCR amplified, cloned, and sequenced from the indicated cell fractions. Zebrafish *izf1* is encoded by eight alternatively spliced exons with four zinc finger DNA binding domains (blue) and two zinc finger dimerization domains (red). Cell fractions included *rag2*:*DsRed* positive, *rag2*:*DsRed;tp53* mutant positive, *rag2*:*EGFP-CA-CaMKII* positive, and *rag2*:*EGFP-CA-CaMKII;tp53* mutant positive B cells. The “x” indicates expression of the particular *ikzf1* splice variant in fluorescently sorted kidney marrow B cells. Ikvar3-7 resulted from frameshifts that induced premature stop codons (S4 Table).

The reduction of productive VDJ rearrangements in *rag2*:*EGFP-CA-CaMKII; tp53* mutant B cells was reminiscent of *ikzf1* mutant fish, where productive VDJ rearrangements are reduced in the *igm* loci and lost in the *igt* loci [57]. IKZF1 is a zinc finger transcription factor that is important for lymphoid differentiation. It is composed of four zinc finger DNA binding domains and two zinc finger dimerization domains [58,59]. Alternative splicing creates several isoforms that can homo- or heterodimerize with IKZF1 or other IKAROS family members (IKZF2-5) [58–61]. However, shorter variants that lack DNA binding activity act in a dominant negative fashion, inhibiting IKZF1 function [58,62]. Therefore, *ikzf1* splice variants were investigated in *rag2*:*DsRed*^+^, *rag2*:*EGFP-CA-CaMKII*^+^, *rag2*:*DsRed; tp53* mutant^+^, and *rag2*:*EGFP-CA-CaMKII; tp53* mutant^+^ B cells. DsRed^+^ kidney marrow B cells from *rag2*:*DsRed* and *rag2*:*DsRed; tp53* mutant fish expressed functional *ikzf1* variants. *Rag2*:*EGFP-CA-CaMKII*^+^ B cells predominantly expressed functional variants, but also expressed two nonfunctional variants generated as a result of incorrect splicing (Ikvar2 and Ikvar5). In addition to reduced expression of *ikzf1* (Fig 6A), *rag2*:*EGFP-CA-CaMKII; tp53* mutant B cells had altered *ikzf1* splicing which induced frameshifts and premature stop codons (Ikvar1-7; Fig 7). Given IKZF1 is frequently mutated or deleted in patients with B ALL [30,62,63], reduced expression and improper splicing of *ikzf1* could promote leukemia development in *rag2*:*EGFP-CA-CaMKII; tp53* mutant fish.

### Pharmacological inhibition of CaMKII alters cell cycle distribution and induces cell death in pre-B ALL cells

**Fig 8:**
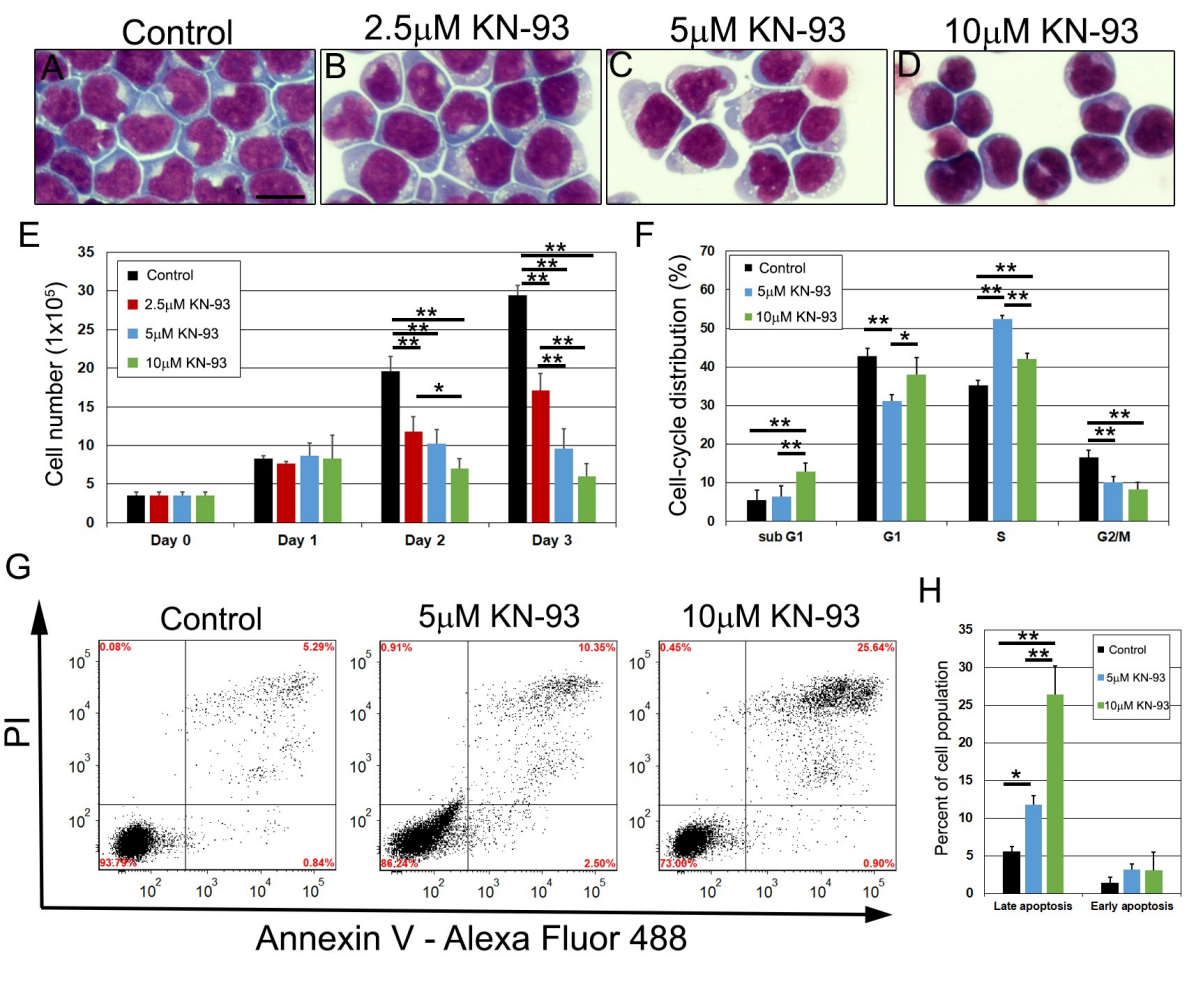
*Pharmacological inhibition of CaMKII induces cell death in human pre-B ALL cells*. HEMA3 stained NALM6 cells treated with 2.5μM, 5μM, and 10μM KN-93 at 48h (A-D). NALM6 growth curves were assessed at 24-hour intervals until 72h after KN-93 treatment. (E, n = 3). Cell cycle distribution was assessed after PI staining in control, 5μM, and 10μM KN-93 treated NALM6 cells at 48h (F, n = 5). NALM6 cells were stained with Annexin V and PI after KN-93 treatment and analyzed using flow cytometry at 48h. The lower left quadrant are cells that are negative for Annexin V and PI, the upper left quadrant is PI positive only indicative of necrosis, the upper right quadrant identifies cells that are positive for both Annexin V and PI indicating late apoptosis, and the bottom right quadrant shows cells that are Annexin V positive, which indicates early stages of apoptosis (G). The bar graph shows the percent of cells that are in early and late stage of apoptosis from four experiments (H). P values were calculated using one-way ANOVA followed by Tukey HSD. * p<0.05 and ** P<0.01. Scale bar: 20 μm in A.

Increased expression and activation of CaMKII has been observed in both cell lines and patient samples of diverse hematological malignancies [21,24,64–66]. Specifically, it was reported that NALM6 cells, a human pre-B ALL cell line, expressed elevated levels of γ CaMKII compared to normal blood as well as high levels of autophosphorylated CaMKII [28,29]. RT-PCR analysis of CaMKII identified expression of cytosolic variants of *CaMK2G* (γ-B, γ-C, γ-E, γ-H) and *CaMK2D* (δ-E) in NALM6 cells. Therefore, NALM6 cells were treated with KN-93, an established CaMKII pharmacological inhibitor, to determine if CaMKII is functionally important in pre-B ALL maturation [17,67,68]. Cells were treated with 2.5μM, 5μM, or 10μM KN-93 and analyzed after HEMA3 staining (Fig 8A–8D). Cell numbers were reduced in KN-93 treated samples and chromatin appeared more condensed (Fig 8D). Time course analysis confirmed cell number occurs in a dose dependent manner beginning at day 2 and continuing through day 3 (Fig 8E). Analysis of cell cycle distribution noted an increase in S phase and a reduction in G1 and G2/M phase distribution with 5μM treatment, and an increase in sub G1 and S phase distribution and reduction in G2/M distribution with 10μM treatment (Fig 8F). Our results indicate that CaMKII inhibition alters cell-cycle distribution and likely induces cell death in a pre-B ALL cell line. Analysis of early and late stage apoptosis using PI and Annexin V (Fig 8G and 8H) indicated that KN-93 did not affect early apoptosis, but significantly promoted late apoptosis (Fig 8H). These results support a role for CaMKII in the proliferation of human pre-B ALL cells in culture.

## Discussion

ALL is the most common childhood cancer and comprises approximately 25% of all pediatric cancers. ALL is most commonly identified in B lineage cells and is diagnosed when lymphoblast composition of the bone marrow or peripheral blood exceeds 25% [54]. Extramedullary involvement with splenomegaly or hepatomegaly occurs in approximately 20% of patients [54]. Our results demonstrate that activated CaMKII on a *tp53* mutant background in zebrafish induces B cell hyperplasia in both the kidney and spleen progressing to an overt leukemia/lymphoma phenotype as early as four months of age, with only 29% of fish surviving the first year of life. Our results indicate that activation of CaMKII can cooperate with *tp53* mutation to drive B lineage malignancy, highlighting the importance of properly regulated Ca^2+^ signaling acting through CaMKII during normal B cell development.

Dysregulation of Ca^2+^ signaling is associated with multiple human disorders including, heart failure, Alzheimer’s, and cancer development in both animal models and patients [11,69,70]. Ca^2+^ is normally transiently elevated to activate downstream signaling molecules, such as CaMKII. Prolonged activation of CaMKII has been linked to diverse malignancies, including leukemia [21,71,72]. Patient samples and cells in culture show increased CaMKII expression as well as increased autophosphorylation of CaMKII at T287, leading to persistent Ca^2+^-independent activity [21,29]. In addition, the tumor suppressor protein phosphatase 2A (PP2A) is often inactivated in hematopoietic malignancies [73–76]. Since, PP2A dephosphorylates CaMKII at T287, returning the holoenzyme to its Ca^2+^-dependent regulatory state [77], inappropriate activation of CaMKII would be an expected result of loss, mutation, or inactivation of PP2A. Furthermore, increased CaMKII activation could result from dysregulation of cytosolic Ca^2+^ concentrations. In leukemia, decreased expression of SERCA3 or increased expression of IP3R2, inhibits maintenance of internal ER Ca^2+^ stores leading to activation of Ca^2+^-dependent signaling molecules [11].

Although expression of CA-CaMKII did not induce leukemia development on its own, increased CaMKII activity was associated with new progression to hematologic malignancy on a *tp53* predisposition background, indicating it functions as an additional “hit” during transformation. Consistent with this role, multiple leukemic cell lines display increased CaMKII expression and autophosphorylation in the presence of background leukemia drivers, such as BCR-ABL [21,78]. In addition, expression of *camk2d1* is increased in immature B lymphoblasts but not T cells of zebrafish with *cmyc*-driven T, B, and mixed lineage leukemia [7]. Furthermore, increased expression of CaMKII was identified in a subset of pediatric patients with B ALL [79,80]. Altogether, these results suggest altered CaMKII expression promotes leukemia transformation on different genetic backgrounds, but is not the primary driver of malignancy. Mutations and deletions in tumor suppressors, such as TP53 and ARF, have been linked to ALL transformation [47,51]. Although *TP53* mutations infrequently occur during the discovery phase of ALL, inactivating TP53 mutations are common upon relapse [47]. In addition, deletions in *ARF* are commonly identified in patients with ALL, with an incidence rate varying from 18–45% [51,81]. ARF signals to TP53 to enable cell cycle arrest or apoptosis after DNA damage [82]. Although an ortholog of ARF has not been identified in zebrafish, TP53 is a key mediator of ARF tumor suppression, and inhibition of the TP53 cellular response likely mimics ARF deletion, as previously reported [52].

Expression of EGFP-CA-CaMKII in lymphocytes of *tp53* mutant zebrafish caused lymphoblast proliferation, similar to ALL. Molecular analysis of leukemic lymphoblasts confirmed B lineage with expression of *rag1*, *rag2*, cd79a, *igiv1s1*, *igic1s1*, *pax5*, *ighm-C*, *and ight-C* and lack of T cell markers *lck* and *tcrd-C*. Immaturity of tumor cells was indicated by blast morphology and expression of *rag1*, which is not expressed in mature, quiescent B cells [43]. Further analysis of EGFP+ leukemic lymphoblasts determined that the expression of the zinc-finger transcription factor, *ikzf1*, was reduced and incorrect splicing induced nonfunctional variants. Ikaros1 (*Ikzf1*) is an essential gene during lymphoid differentiation and is often deleted or mutated in patients with B ALL [2,30,31]. In zebrafish, mutations in *ikzf1* leads to lower thymopoiesis efficiency [57,83], loss of kidney marrow B cell *igt* rearrangements and reduced productive *igm* rearrangements [57]. Zebrafish *ikzf1* mutants do not develop overt leukemia or lymphoma, likely requiring an additional genetic mutation or alteration [57,83]. Similar to *ikzf1* mutants, *rag2*:*EGFP-CA-CaMKII; tp53* mutant B cells had reduced productively rearranged *igm* and *igt* alleles. Genomic VDJ recombination is essential for maturation of the adaptive immune system. Without productive rearrangements, the immune system is compromised which could lead to chromosomal rearrangements and B cell malignancies [84]. The limited number of productive IgH rearrangements in *rag2*:*EGFP-CA-CaMKII; tp53* mutant fish could reduce the effectiveness of the adaptive immune system. Leukemic fish could therefore be susceptible to infections, leading to lethargy and death. Thus, reduced expression and frameshift mutations of *ikzf1* in *rag2*:*EGFP-CaMKII; tp53* mutant kidney marrow B lymphoblasts could lead to reduced productive VDJ recombination and thus inhibit B cell maturation, promoting leukemia.

Although *rag2*-driven oncogenes in previous zebrafish models predominantly generated T-ALL [7,8], our model did not express T cell lineage markers. The specific effect on B cells is likely due to the combinatorial effect of increased activation of CaMKII and the *tp53* mutant background. This is the first study to show a role for activated CaMKII in B ALL using a model system and the second zebrafish B ALL model driven by *rag2* [7]. Therefore, this model provides novel insight into the role of calcium signaling in leukemia maturation and can be used to better understand the role of CaMKII in B ALL.

## Materials and methods

### Ethics statement

All animal research was conducted under approved IACUC protocols, and in compliance with the Institutional Animal Care and Use Committee (IACUC) of Virginia Commonwealth University and according to the American Veterinary Medical Association (AVMA) guidelines.

### Zebrafish husbandry and transgenic lines

Wild type (AB), *Tg(rag2*:*GFP)*^*zdf8*^ [8], Tg(Rag2:DsRed)^zf411^ [38] and *tp53*^*zdf1*^ [53] mutant lines were raised as previously described [13,16–18]. Fish were not selected based on sex and no randomization was used in this study.

### Transgenic line generation

Gateway cloning (Invitrogen) was used to produce a destination transgenesis construct with the zebrafish *recombination activating gene 2* (*rag2*) promoter [8] driving expression of a constitutively active (CA, T287D) CaMKII [13,16], flanked by Tol2 transposase cis targeting elements [3]. 25 ng of *rag2*:*EGFP-caHsa*.*CAMK2_T287D* was co-injected with 25 ng of *Tol2* transposase mRNA into AB embryos at the one-cell stage. P0 fish were raised and outcrossed to generate stable F1 *Tg(rag2*:*EGFP-caHsa*.*CAMK2_T287D)* animals (referred to as *rag2*:*EGFP-CA-CaMKII* in the text). F2 stable transgenics were crossed into the *tp53* mutant background to generate a stable line carrying both transgene and mutant alleles. Transgene copy number was not determined in *rag2*:*EGFP-CA-CaMKII; tp53* wild type or mutant fish since EGFP brightness was not discernible between heterozygote and homozygote fish. The functionality of the T287D mutation was previously demonstrated in both cells in culture and zebrafish embryos [13,16,33].

### Flow cytometry

Zebrafish kidneys and spleens were dissected into 1X PBS + 4% FBS, passed through a 40 μm filter, centrifuged at 800 rpm for 5 minutes, and washed twice in 1X PBS + 4% FBS [8]. Cell populations were incubated with DAPI to eliminate dead cells and analyzed on a Fortessa (Becton-Dickinson, B-D) analyzer or sorted on a FACS Aria II or Fusion Aria high-speed analyzer/sorter (B-D) and saved in either TRIzol reagent (Invitrogen) or 1X PBS + 4% FBS.

### Pathology and immunohistochemistry

Fish were fixed in 10% buffered formalin for 3 days and decalcified in 0.5M EDTA. Fixed tissue was prepared for paraffin infiltration using a Tissue Tek VIP5 Processor. Paraffin embedded tissue was sectioned at 5 μm, floated into a 40°C water bath, and mounted on positively charged slides. Slides were baked for 15 minutes at 60°C. Hematoxylin and eosin (H&E) staining followed wax removal in Xylene and re-hydration through ethanol. Slides were imaged on a Nikon Eclipse E600 microscope using Elements AR 3.10 software.

Paraffin embedded sections were processed for immunofluorescence using a BOND RX strainer (Leica). Sections were blocked in 5% NGS for 20 min and incubated with rabbit anti-GFP (Thermo Fisher, CAB4211) for 30 minutes. Slides were imaged as above.

### cDNA synthesis and PCR

Lymphoid populations were flow sorted into TRIzol reagent (Invitrogen), total RNA was isolated, and cDNA was synthesized as previously described [13,14,16]. PCR was performed using gene-specific primers (S1 Table) [4,43,85]. PCR primers that bracket the variable region of *camk2* were used to amplify fragments that were cloned into the Strataclone vector (Agilent) for sequencing, as previously described [13,14] (S4 Table). Zebrafish *ikzf1* was PCR amplified using primers that amplify sequence from exon2 to exon8 (S1 Table), cloned using the Strataclone PCR cloning kit (Agilent), and sequenced to identify *ikzf1* splice variants from fluorescently sorted lymphocytes (S5 Table).

NALM6 cells were harvested, washed in 1X PBS, and total RNA was isolated using TRIzol (Invitrogen) reagent. cDNA was synthesized and used as the template for PCR amplification of δ and γ camk2 genes (S1 Table). Amplified products were cloned into the Strataclone (Agilent) vector for sequencing.

### Genomic isolation and PCR

Kidney marrow cells were harvested into DNA lysis buffer and genomic DNA was isolated as previously described [86]. Semi-nested PCR was then completed using the following protocol: denaturation at 94°C for 3 minutes, followed by 30 cycles of 94°C 30 seconds, 53°C 30 seconds, 72°C 60 seconds, and a final extension cycle of 72°C for 10 minutes. Cleaned-up samples were used as a template in the second PCR reaction: denaturation at 94°C for 3 minutes, followed by 30 cycles of 94°C 30 seconds, 58°C 30 seconds, 72°C 60 seconds, and a final extension cycle of 72°C for 10 minutes [87]. *Ef1a* served as a control.

### Immunofluorescence

Zebrafish kidneys and spleens were prepared the same as flow cytometry. Filtered cells were then cytospun on a Cytospin2 (Shandon) at 300 rpm for 3 minutes onto glass cover slips. Cells were fixed in 4% formaldehyde for 10 minutes, washed three times in 1X PBS, blocked in 5% bovine serum albumin/10% normal goat serum for 30 minutes, and incubated in rabbit anti-GFP (Thermo Fisher, CAB4211) primary antibody for one hour at 37°C. Cells were washed three times in 1X PBS and incubated in goat-anti-rabbit Alexa-488 (Invitrogen) secondary antibody. Cover slips were mounted on glass slides (Fisher) and imaged on a Nikon C2 laser scanning confocal on a Nikon Eclipse Ni microscope using Elements AR 4.50 software.

### Blood staining

Filtered kidney marrow and spleen were cytospun at 300 rpm for 3 minutes onto glass slides. Cells were stained using HEMA3 (Fisher) and imaged on a Nikon Eclipse E600 microscope.

### High-speed video microscopy

Live adults were imaged using epifluorescence after transient anesthesia. EGFP positive lymphocytes were imaged in the caudal fin using a Nikon AZ100 Stereomicroscope at 20 frames per second [18].

### Confocal microscopy

*Rag2*:*DsRed; rag2*:*EGFP-CA-CaMKII* embryos were anesthetized at 4 dpf and mounted on chamber slides, then imaged on a Nikon C2 laser scanning confocal on a Nikon Eclipse Ni microscope using Elements AR 4.50 software.

### Cell lines and culture

NALM6 human lymphocyte-like cells (CRL 3273, DUX4-IGH translocation) were acquired from American Type Culture Collection (ATCC). Cells were suspended in polystyrene flasks containing RPMI-1640 medium (Gibco), supplemented with 10% fetal bovine serum (Sigma Aldrich), Glutamax (Gibco) and penicillin/streptomycin (Gibco). Cells were cultured in a humidified chamber with 5% CO_2_ at 37°C to log phase before subculturing every 3–4 days for no more than 15 passages.

### Cell number analysis

NALM6 cells were grown in 6-well dishes and treated with varying concentration of water-soluble KN-93 (Calbiochem, Fisher Scientific). The range of KN-93 used in this study is identical to that which was previously shown to competitively inhibit both endogenous mammalian and zebrafish CaMKII activity and cell behaviors [15,17,67,68,88]. Cell number was assessed at 24-hour intervals until 72hpf using the Countess 3 Automated Cell Counter (Thermo Fisher).

### Cell cycle analysis

NALM6 cells were treated and grown in 6-well dishes (Falcon) containing different concentration of KN-93 (Calbiochem, Fisher Scientific) for 48h and prepared as previously described [89]. Samples were assayed using a Fortessa (B-D) analyzer.

### Cell death analysis

NALM6 cells were grown in 6-well dishes (Falcon) with varying concentrations of KN-93 for 48h and assayed for apoptosis using Alex Fluor-488 Annexin V (Thermo Fisher) and PI (Sigma Aldrich). Samples were assayed using a Fortessa (B-D) analyzer.

### Statistical analysis

Statistical analyses were conducted using one-way ANOVA followed by Tukey’s HSD for flow cytometry (S2 Table) and blood smears. Survival curve statistical significance was determined using the Log-rank (Mantel-Cox) test and was confirmed using the Gehan-Breslow-Wilsoxon test using GraphPad Prism 9.5.1. IgH VDJ recombination statistical significance was calculated using Fisher’s exact test. Center values are calculated as the mean and error bars are standard deviations.

## Supporting information

S1 Table List of PCR primers used in this study. (DOCX)

S2 Table FACS statistical analysis. P values of sorted kidney marrow cells from *rag2*:*EGFP-CA-CaMKII; tp53* wild type, *tp53* mutant, and *rag2*:*EGFP-CA-CaMKII; tp53* mutant fish compared to *rag2*:*GF*P fish were calculated using one-way ANOVA followed by Tukey HSD. Statistically significant results (P<0.05) are shown in bold.
(DOCX)

S3 Table Genomic DJ rearrangements. Sequenced *igm* and *igt* genomic DJ rearrangements from *rag2*:*EGFP-CA-CaMKII*, *tp53* mutant, and *rag2*:*EGFP-CA-CaMKII; tp53* mutant kidney marrow B cells.
(DOCX)

S4 Table Alternative *camk2* splice variants identified in wild type kidney marrow lymphocytes. RT-PCR was performed using primers that flank the variable domain and the products were cloned and sequenced to identify *camk2* splice variants. Variable domain exons are identified by underlined red or black font. Catalytic domain sequence is N-terminal and association domain is C-terminal to the variable domain sequence in black.
(DOCX)

S5 Table Alternative *ikzf1* splice variants identified in *rag2*:*EGFP-CA-CaMKII; tp53* mutant positive kidney marrow lymphocytes as a result of incorrect alternative splicing. RT-PCR using primers that bind to exon2 and exon8, products cloned, and sequenced to identify *ikzf1* splice variants. Exons are identified by red or black font. Incorrectly spliced sequence is denoted in blue.
(DOCX)

S1 Fig Splenomegaly in a subset of leukemic fish. (A,B) Spleen (outlined by dashed lines) size in *rag2*:*EGFP-CA-CaMKII* transgenic and *tp53* mutant animals is normal compared to the enlarged spleen seen in (C) *rag2*:*EGFP-CA-CaMKII; tp53* mutants. Head kidney (arrow) is also pictured.
(TIF)

S2 Fig Nonhematopoietic tissue is normal in *rag2*:*EGFP-CA-CaMKII; tp53* mutant fish. Histological sections are normal in *rag2*:*EGFP-CA-CaMKII* wild type, *tp53* mutant, and *rag2*:*EGFP-CA-CaMKII; tp53* mutant fish for (A-C) thymus, (D-F) gills, (G-I) muscle, and (J-L) liver.
(TIF)

S1 Data Index of supplementary data tables by order of appearance. (XLSX)

S1 Video EGFP positive lymphocytes circulating in the caudal fin of *rag2*:*EGFP-CA-CaMKII* fish. (MP4)

S2 Video EGFP positive lymphocytes circulating in the caudal fin of *rag2*:*EGFP-CA-CaMKII; tp53* mutant fish. (MP4)
