# Supplementary material for: Constitutively active CaMKII Drives B lineage acute lymphoblastic leukemia/lymphoma in tp53 mutant zebrafish
Source: PLoS Genet. 2023 Dec 20;19(12):e1011102. doi: 10.1371/journal.pgen.1011102 (PMC10766190; doi:10.1371/journal.pgen.1011102)
Supplement: S1 Table — (DOCX) [file pgen.1011102.s001.docx]

**S1 Table List of PCR primers used in this study.**

| **Primer** | **Sequence** |
| --- | --- |
| *S rag2* | ACGCTCATGTCCAACTGGGATAT |
| *AS rag2* | TTGAGGCGGACAGTCACCTACACT |
| *S lck* | AGATGAATGGTGTGACCAGTGTA |
| *AS lck* | GATCCTGTAGTGCTTGATGATGT |
| *S igiv1s1* | GTTCCTGACCAGTGCAGAGA |
| *AS igiv1s1* | CCTGATCACCTCCAGCATGA |
| *S cd79a* | GAAAATGGGGTTCAGGCTGGTGC |
| *AS cd79a* | GCTACGGCTTCTCCAGCTGAATGTC |
| *S ighm-C* | GATTTGAGCGATTTTGTGCAGTACCC |
| *AS ighm-C* | CGGTTTGTCAAAGGTTCTTCTTTCTC |
| *S rag1* | AAGAACCAGGTGAAGACATTTGCC |
| *AS rag1* | TGCCACATCATAACGGAATCGTC |
| *S ight-C* | AACGTCACCCAGCATTCTACAGC |
| *AS ight-C* | CTTTGTTATTAAGCGTGTCC |
| *S ikzf1 exon1* | CAGGATGGAGACTGAGGAGGCACAG |
| *AS ikzf1 exon8* | TCAGATGCGGTGCTCTCCACG |
| *S ikzf1 exon2* | CCAACACAACAATCGCACAG |
| *S igic1s1* | GGCACCAAACTGGATGTTG |
| *AS igic1s1* | AGCAGGAGTGTGTGTGTGCT |
| *S pax5* | CACGGCATGGACTTGGACCAAAAG |
| *AS pax5* | CGATCGTAAGCACTGGCAGTTGC |
| *S tcrd-C* | AAGAGACAGTGAATTCTCCTC |
| *AS tcrd-C* | TGATTACAAAGATCAGAGCCTC |
| *S ef1a* | GTGCTGTGCTGATTGTTGCT |
| *AS ef1a* | TGTATGCGCTGACTTCCTTG |
| *S human Camk2* | CGCTCCGGAACAAGGAATTTCTCAGCAGCCAA |
| *AS human Camk2* | cccggatcctcactgcagcggtgcggcag |
| S IgVH1-Jtm (first) | gatggacgtgttacaatttgg |
| S IgVH1-Jtm (second) | CCTCCTCAGACTCTGTGGTGA |
| S IgVH2-Jtm (first) | TTGTAACATGACCATGAATATT |
| S IgVH2-Jtm (second) | CGATTAGATCAGTCACCTTCT |
| S IgVH3-Jtm (first) | CATGACAATGGATATTGTGTCC |
| S IgVH3-Jtm (second) | CTCTGTTGGTGTCAAACACTG |
| AS IgVH-Jt | AAGGTCTATTACTAACAGATCAC |
| AS IgVH-Jm | GTTCCCTTTCCCCAGTAGTCAAA |
