# Supplementary material for: Constitutively active CaMKII Drives B lineage acute lymphoblastic leukemia/lymphoma in tp53 mutant zebrafish
Source: PLoS Genet. 2023 Dec 20;19(12):e1011102. doi: 10.1371/journal.pgen.1011102 (PMC10766190; doi:10.1371/journal.pgen.1011102)
Supplement: S2 Table — P values of sorted kidney marrow cells from rag2:EGFP-CA-CaMKII; tp53 wild type, tp53 mutant, and rag2:EGFP-CA-CaMKII; tp53 mutant fish compared to rag2:GFP fish were calculated using one-way ANOVA followed by Tukey HSD. Statistically significant results (P<0.05) are shown in bold. (DOCX) [file pgen.1011102.s002.docx]

**S2 Table. FACS statistical analysis.**

| **Kidney Marrow** | **Erythroid** | **Lymphoid** | **Myeloid** | **Precursor** | **GFP** |
| --- | --- | --- | --- | --- | --- |
| ***rag2:GFP*** | NA | NA | NA | NA | NA |
| ***rag2:EGFP-CA-CaMKII*** | 0.889 | 0.436 | 0.592 | 0.898 | 0.887 |
| ***tp53* mutant** | 0.889 | 0.899 | 0.845 | 0.823 | NA |
| ***rag2:EGFP-CA-CaMKII; tp53* mutant** | 0.429 | **0.029** | 0.750 | 0.243 | **0.001** |
|  |  |  |  |  |  |
|  |  |  |  |  |  |
