# Supplementary material for: Constitutively active CaMKII Drives B lineage acute lymphoblastic leukemia/lymphoma in tp53 mutant zebrafish
Source: PLoS Genet. 2023 Dec 20;19(12):e1011102. doi: 10.1371/journal.pgen.1011102 (PMC10766190; doi:10.1371/journal.pgen.1011102)
Supplement: S3 Table — Sequenced igm and igt genomic DJ rearrangements from rag2:EGFP-CA-CaMKII, tp53 mutant, and rag2:EGFP-CA-CaMKII; tp53 mutant kidney marrow B cells. (DOCX) [file pgen.1011102.s003.docx]

**S3 Table. Genomic DJ rearrangements.**

| **IGM1** |  |  |  |  |
| --- | --- | --- | --- | --- |
| ***tp53* mutant** | |  | **PRODUCTIVE** | **NON-PRODUCTIVE** |
| 1 | CAREMEWRSTTLTTGERE…* |  |  | 2 |
| 2 | CARDFNYWAFDYWGKGT |  | 1 |  |
| 3 | CARSIAGYFDYWGKGT |  | 1 |  |
| 4 | CARTSLGPFDYWGKGT |  | 1 |  |
| 5 | CARTDSGGYFDYWGKGT |  | 1 |  |
| 6 | CARTGVYYYFDYWGKGT |  | 1 |  |
| 7 | CARTQWRAFDYWGKGT |  | 1 |  |
| 8 | CARTLTTMLLTTGE…* |  |  | 2 |
| 9 | CARDGVAWSATPRHKLS* |  |  | 1 |
| 10 | CARQYTWAFDYWGKGT |  | 1 |  |
| 11 | CAREMEWHYFDYWGKGT |  | 1 |  |
| 12 | CARDDNYCFDYGERE |  |  | 1 |
| 13 | CARDDEYCYFDFCFDERGKGT |  | 1 |  |
| 14 | CARDDNYGARDYWGKGT |  | 1 |  |
| 15 | RASGDAFDYWGKGT |  | 1 |  |
| 16 | CARTRAEYYYFDYWGKGT |  | 1 |  |
| 17 | CARSGVLRLLL* |  |  | 1 |
| 18 | CARTEWGTLTTGERE…* |  |  | 1 |
| 19 | CARTNNGAFDYWGKGT |  | 1 |  |
|  |  | **Total** | **13** | **8** |
|  |  |  |  |  |
| ***rag2:EGFP-CA-CaMKII; tp53* mutant** | |  | **PRODUCTIVE** | **NON-PRODUCTIVE** |
| 1 | CARDSGYAFDYWGKGT |  | 2 |  |
| 2 | CARTGWDFDFFFDYWGKGT |  | 4 |  |
| 3 | CAR* |  |  | 3 |
| 4 | CARE* |  |  | 1 |
| 5 | CARTV* |  |  | 1 |
| 6 | CARTDNAFDYWGKGT |  | 1 |  |
| 7 | CARDEWRYYFDYWGKGT |  | 1 |  |
| 8 | CARTSPAGAAFDYWGKGT |  | 1 |  |
| 9 | CARDGVELLRLLL* |  |  | 1 |
| 10 | CARTKSYYYFDYWGKGT |  | 1 |  |
| 11 | CARDLYGVLTTGERE…* |  |  | 2 |
| 12 | CARD* |  |  | 1 |
| 13 | CARDYGDAFDYWKGKGT |  | 1 |  |
| 14 | CARTGLLTTGERE…* |  |  | 1 |
|  |  | **Total** | **11** | **10** |
|  |  |  |  |  |
|  |  |  |  |  |
| ***rag2:EGFP-CA-CaMKII; tp53* WT** | |  | **PRODUCTIVE** | **NON-PRODUCTIVE** |
| 1 | CARTYNGAFDYWGRG |  | 3 |  |
| 2 | CARMEWLHFDYWGKGT |  | 1 |  |
| 3 | CARGYNNYFDYWGKGT |  | 2 |  |
| 4 | CARNNYNAFDYWGKGT |  | 2 |  |
| 5 | CARTGHYDFDYWGKGT |  | 1 |  |
| 6 | CARNSGSNWAFDYWGKGT |  | 1 |  |
| 7 | CARTINYGAFDYWGKGT |  | 5 |  |
| 8 | CARLAYGAFDYWKGKGT |  | 1 |  |
| 9 | CARGYDYFDYWGKGT |  | 1 |  |
| 10 | CARDDNNAFDYWGKGT |  | 1 |  |
| 11 | CARDSDYFDYWGKGT |  | 1 |  |
| 12 | CARTNGVADYFDYWGKGT |  | 1 |  |
| 13 | CARTGHNAFDYWGKGT |  | 1 |  |
|  |  | **Total** | **21** | **0** |
|  |  |  |  |  |
|  |  |  |  |  |
|  |  |  |  |  |
| **IGM2** |  |  |  |  |
| ***tp53* mutant** | |  | **PRODUCTIVE** | **NON-PRODUCTIVE** |
| 1 | RAREPYNGAFDYWKGKGT |  | 3 |  |
| 2 | CARDRIRGALLTTGERE…* |  |  | 1 |
| 3 | CAREPNYGAFDYWGKGT |  | 2 |  |
| 4 | CAREPLYGCLLTTGER…* |  |  | 1 |
| 5 | CAREPLYGYSAFDYWGKGT |  | 2 |  |
| 6 | CANNGAFDYWGKGT |  | 6 |  |
| 7 | CARENNNAFDYWGKGT |  | 1 |  |
| 8 | CAREPYNNYGWAFDYWGKGT |  | 1 |  |
| 9 | CAREPGDGGDAFDYWGKGT |  | 3 |  |
| 10 | CARPNYGAFDYWGKGT |  | 1 |  |
| 11 | CAREPGSSGAFDYWGKGT |  | 1 |  |
|  |  | **Total** | **20** | **2** |
|  |  |  |  |  |
| ***rag2:EGFP-CA-CaMKII; tp53* mutant** | |  | **PRODUCTIVE** | **NON-PRODUCTIVE** |
| 1 | CARSGGLL* |  |  | 13 |
| 2 | CAREPYNRGAFDYWKGKGT |  | 2 |  |
| 3 | CARDSGFYYFDYWGKGT |  | 1 |  |
| 4 | CARELTTTYAFDYWGKGT |  | 2 |  |
| 5 | CAREPGSVPGAFDYWGKGT |  | 2 |  |
| 6 | CAREP* |  |  | 1 |
| 7 | CARDEISYFDFFFDYWGKGT |  | 1 |  |
| 8 | CAREPGVRGGAAFDYWGKGT |  | 1 |  |
| 9 | CAREK* |  |  | 1 |
| 10 | CAREPGYGVALL* |  |  | 1 |
| 11 | CAREPGEWRTTLTTGERE…* |  |  | 5 |
|  |  | **Total** | **9** | **21** |
|  |  |  |  |  |
| ***rag2:EGFP-CA-CaMKII; tp53* WT** | |  | **PRODUCTIVE** | **NON-PRODUCTIVE** |
| 1 | CAREYPNAFDYWGKGT |  | 11 |  |
| 2 | CAREPGGDYFDFFFDYWGKGT |  | 4 |  |
| 3 | CAREPYNAFDYWGKGT |  | 1 |  |
| 4 | CARELTTTYAFDYWGKGT |  | 1 |  |
| 5 | CAREPGTGWGPLRLLL* |  |  | 3 |
| 6 | CAREPGGLGGAIFFDYWGKGT |  | 1 |  |
| 7 | CAPIRGSCF* |  |  | 1 |
|  |  | **Total** | **18** | **4** |
|  |  |  |  |  |
|  |  |  |  |  |
|  |  |  |  |  |
| **IGM3** |  |  |  |  |
| ***tp53* mutant** | |  | **PRODUCTIVE** | **NON-PRODUCTIVE** |
| 1 | CAREYDGAFDYWGKGT |  | 3 |  |
| 2 | CAKQYNNAFDYWGKGT |  | 1 |  |
| 3 | CARRSGYFDYWGKGT |  | 2 |  |
| 4 | CARVRGPSYFDFFFDYWGKGT |  | 1 |  |
| 5 | CARVPNYYGAFDYWGKGT |  | 4 |  |
| 6 | CARVLRCL* |  |  | 1 |
| 7 | CAGDSGSMLLTTGERE…* |  |  | 1 |
| 8 | CARDSNYDNAFDYWGKGT |  | 1 |  |
| 9 | CARGGLGWPAFDYWGKGT |  | 2 |  |
| 10 | CAREGGGFDYWGKGT |  | 1 |  |
| 11 | CARSLGYFDFFFDYWGKGT |  | 1 |  |
| 12 | CARITTSYFDYWGKGT |  | 1 |  |
| 13 | CARVGEWRAFDYWGKGT |  | 1 |  |
| 14 | CVRVLYYFDYWGKGT |  | 1 |  |
| 15 | CAMECNAFDYWGKGT |  | 1 |  |
|  |  | **Total** | **20** | **2** |
|  |  |  |  |  |
| ***rag2:EGFP-CA-CaMKII; tp53* mutant** | |  | **PRODUCTIVE** | **NON-PRODUCTIVE** |
| 1 | CARV* |  |  | 4 |
| 2 | CAREGGGFDYWGKGT |  | 1 |  |
| 3 | CARRSGDYFDYWKGKGT |  | 1 |  |
| 4 | CARQSNNAFDYWGKGT |  | 1 |  |
| 5 | CARITTSYFDYWGKGT |  | 1 |  |
| 6 | CTRQLRE* |  |  | 1 |
| 7 | CARVLRCL* |  |  | 1 |
| 8 | CARSLGYFDFFFDYWGKGT |  | 1 |  |
| 9 | CARVPNYYGAFDYWKGKGT |  | 1 |  |
| 10 | CARAGSYDFFFDYWGKGT |  | 1 |  |
| 11 | CARMEWYYFDYWGKGT |  | 1 |  |
| 12 | CAVTTWAFDYWGKGT |  | 1 |  |
| 13 | CARGSTINAFDYWGKGT |  | 1 |  |
| 14 | CARFLTTTLTTGER…* |  |  | 1 |
| 15 | CARAITGNYFDYWGKGT |  | 1 |  |
| 16 | CASRYFDYWGKGT |  | 1 |  |
| 17 | CARGANNYGAFDYWGKGT |  | 3 |  |
| 18 | CARVDSGGRAFDYWGKGT |  | 1 |  |
|  |  | **Total** | **16** | **7** |
|  |  |  |  |  |
| ***rag2:EGFP-CA-CaMKII; tp53* WT** | |  | **PRODUCTIVE** | **NON-PRODUCTIVE** |
| 1 | CARVQVGCF* |  |  | 4 |
| 2 | CARAAIRGGLSLLRLLL* |  |  | 1 |
| 3 | CARPNYGARDYWGKGT |  | 2 |  |
| 4 | CARYNYYGAFDYWGKGT |  | 2 |  |
| 5 | CARFPQYNNAFDYWGKGT |  | 2 |  |
| 6 | CARV* |  |  | 3 |
| 7 | CA* |  |  | 2 |
| 8 | CARGRDGAFDYWGKGT |  | 1 |  |
| 9 | CARVLRDAFDYWGKGT |  | 1 |  |
| 10 | CARGRGTLLLTTGER…* |  |  | 2 |
| 11 | CARPSRGGYFDYWGKGT |  | 1 |  |
| 12 | CARVTTWAFDYWGKGT |  | 1 |  |
| 13 | CARGGGGNAFDYWGKGT |  | 1 |  |
|  |  | **Total** | **11** | **12** |
|  |  |  |  |  |
|  |  |  |  |  |
|  |  |  |  |  |
| **IGT1** |  |  |  |  |
| ***tp53* mutant** | |  | **PRODUCTIVE** | **NON-PRODUCTIVE** |
| 1 | CARTTGWGYFDFFFDYWGRGTQVTV |  | 1 |  |
| 2 | CARLRGLLLTTGVKAKIK |  |  | 2 |
| 3 | CAREGCFDFFFDYWGRGTQVTV |  | 8 |  |
| 4 | CARSVFFTGWGGYFDFFFDYWGRGAQVTV |  | 6 |  |
| 5 | CARDGAWMSYFDYWGRGTQVTV |  | 1 |  |
| 6 | CAREGCLDFFFDYWGRGTQVAVT |  | 1 |  |
| 7 | CARSVFFTGWGELLRLLL* |  |  | 1 |
|  |  | **Total** | **17** | **3** |
|  |  |  |  |  |
| ***rag2:EGFP-CA-CaMKII; tp53* mutant** | |  | **PRODUCTIVE** | **NON-PRODUCTIVE** |
| 1 | CARGYGVGRLLRLLL* |  |  | 6 |
| 2 | CARPGMNFDFFFDYWGRGTQVTV |  | 9 |  |
| 3 | CARDVWDFDFFFDYWGRGTQVTV |  | 2 |  |
| 4 | CARDGKATSTSSLTTGVEGRR* |  |  | 5 |
| 5 | CARPGMELLRLLL* |  |  | 1 |
|  |  | **Total** | **11** | **12** |
|  |  |  |  |  |
|  |  |  |  |  |
|  |  |  |  |  |
| **IGT2** |  |  |  |  |
| ***tp53* mutant** | |  | **PRODUCTIVE** | **NON-PRODUCTIVE** |
| 1 | CAREPGRYFDFFFDYWGRGT |  | 8 |  |
| 2 | CARESRLSYFDFFFDYWGRGT |  | 4 |  |
| 3 | CAREPGRGENYFDYWGRGTQVTVT |  | 3 |  |
| 4 | CAREPGWNFDFFFDYWGRGTQVTV |  | 3 |  |
| 5 | AREPSYTG* |  |  | 1 |
|  |  | **Total** | **18** | **1** |
|  |  |  |  |  |
| ***rag2:EGFP-CA-CaMKII; tp53* mutant** | |  | **PRODUCTIVE** | **NON-PRODUCTIVE** |
| 1 | CAREPPGMGLRLLL* |  |  | 1 |
| 2 | CAREPGWNFDFFFDYWGRGT |  | 5 |  |
| 3 | CAREPGGLGGAIFFDYWGRGTQVTV |  | 10 |  |
| 4 | CAREPGMDFDFFFDYWGRGTQVTVT |  | 1 |  |
| 5 | CAREPGRYFDFFFDYWGRGTQVTV |  | 3 |  |
| 6 | CAREPHTGDFDFFFDYWGRGTQVTV |  | 1 |  |
|  |  | **Total** | **20** | **1** |
|  |  |  |  |  |
|  |  |  |  |  |
|  |  |  |  |  |
| **IGT3** |  |  |  |  |
| ***tp53* mutant** | |  | **PRODUCTIVE** | **NON-PRODUCTIVE** |
| 1 | CASGSYFDFFFDYNGGGTQVTV |  | 8 |  |
| 2 | CARVSYFDFFFDYWGRGTQVTV |  | 10 |  |
| 3 | CASVGSYFDFFFDYWGRGTQVTVT |  | 2 |  |
|  |  | **Total** | **20** | **0** |
|  |  |  |  |  |
| ***rag2:EGFP-CA-CaMKII; tp53* mutant** | |  | **PRODUCTIVE** | **NON-PRODUCTIVE** |
| 1 | CARVPGYFDFFFDYWGRGTQVTV |  | 4 |  |
| 2 | CARVYTGWELRLLL* |  |  | 1 |
| 3 | CARSLGWPLRLLL* |  |  | 1 |
| 4 | CARLYGVLATSTSSLTTGVEGRR* |  |  | 2 |
| 5 | CARGGYFDFFFDYWGRGTQVTVT |  | 1 |  |
| 6 | CAR* |  |  | 1 |
| 7 | CARVYGVGATSTSSLTTGVEGRR* |  |  | 9 |
|  |  | **Total** | **5** | **14** |
