# Supplementary material for: Constitutively active CaMKII Drives B lineage acute lymphoblastic leukemia/lymphoma in tp53 mutant zebrafish
Source: PLoS Genet. 2023 Dec 20;19(12):e1011102. doi: 10.1371/journal.pgen.1011102 (PMC10766190; doi:10.1371/journal.pgen.1011102)
Supplement: S4 Table — RT-PCR was performed using primers that flank the variable domain and the products were cloned and sequenced to identify camk2 splice variants. Variable domain exons are identified by underlined red or black font. Catalytic domain sequence is N-terminal and association domain is C-terminal to the variable domain sequence in black. (DOCX) [file pgen.1011102.s004.docx]

**S4 Table Alternative *camk2* splice variants identified in wild type kidney marrow lymphocytes.**

**Camk2b1 - C**

WICQRSTVASMMHRQETVECLKKFNARRKLKGAILTTMLVSRNFS**AAKTLLNKKADVKESSDSSNATVEDEEMK**ARKQEIIKITEQLIEAINNGDFEAYAKICDPGLTCFEPEALGNLVEGMDFHRFYFENLLSKNSKPIHTTILNPHVHLIGE

**Camk2d2 - E**

WICQRSTVASMMHRQETVECLKKFNARRKLKGAILTTLLVTRNFS**AAKSLLNKKPDGVKEPQTTVIHNPVDRNKESTESANTTIEDEDLK**ARKQEIIKVTEQLIESINNGDFEAYAKICDPGLTSFEPEALGNLVEGHDFHRFYFENALSKGNKPVHTILLNPHVHLIGE

**Camk2b1 – K**

WICQRSTVASMMHRQETVECLKRFNARRKLKGAILTTMLVSRNFS**AAKTLLNKKADVKKRKSSSTIQYMESSDSSNATVEDEEMK**ARKQEIIKITEQLIEAINNGDFEAYAKICDPGLTCFEPEALGNLVEGMDFHRFYFENLLSKNSKPIHTTILNPHVHLIGE

**Camk2g2 - K**

WICQRSTVASMMHRQETVECLRKFNARRKLKGAILTTMLVSRNFS**ACKSLLNKKSDGVKKRKSSSSVYLMGSTESCNTTEEEDMKGRK**ARKQEIIKITEQLIEAINNGDFEAYTRICDPGLTSFEPEALGNLVEGMDFHKFYFENLLSKNSKPVHTTILNPHVHLIGE

**S4 Table. Alternative *camk2* splice variants identified in wild type kidney marrow lymphocytes.**
