# Supplementary material for: Constitutively active CaMKII Drives B lineage acute lymphoblastic leukemia/lymphoma in tp53 mutant zebrafish
Source: PLoS Genet. 2023 Dec 20;19(12):e1011102. doi: 10.1371/journal.pgen.1011102 (PMC10766190; doi:10.1371/journal.pgen.1011102)
Supplement: S5 Table — RT-PCR using primers that bind to exon2 and exon8, products cloned, and sequenced to identify ikzf1 splice variants. Exons are identified by red or black font. Incorrectly spliced sequence is denoted in blue. (DOCX) [file pgen.1011102.s005.docx]

**S5 Table Alternative *ikzf1* splice variants identified in *rag2:EGFP-CA-CaMKII; tp53* mutant positive kidney marrow lymphocytes as a result of incorrect alternative splicing.**

**Camk2b1 - C**

WICQRSTVASMMHRQETVECLKKFNARRKLKGAILTTMLVSRNFS**AAKTLLNKKADVKESSDSSNATVEDEEMK**ARKQEIIKITEQLIEAINNGDFEAYAKICDPGLTCFEPEALGNLVEGMDFHRFYFENLLSKNSKPIHTTILNPHVHLIGE

**Camk2d2 - E**

WICQRSTVASMMHRQETVECLKKFNARRKLKGAILTTLLVTRNFS**AAKSLLNKKPDGVKEPQTTVIHNPVDRNKESTESANTTIEDEDLK**ARKQEIIKVTEQLIESINNGDFEAYAKICDPGLTSFEPEALGNLVEGHDFHRFYFENALSKGNKPVHTILLNPHVHLIGE

**Camk2b1 – K**

WICQRSTVASMMHRQETVECLKRFNARRKLKGAILTTMLVSRNFS**AAKTLLNKKADVKKRKSSSTIQYMESSDSSNATVEDEEMK**ARKQEIIKITEQLIEAINNGDFEAYAKICDPGLTCFEPEALGNLVEGMDFHRFYFENLLSKNSKPIHTTILNPHVHLIGE

**Camk2g2 - K**

WICQRSTVASMMHRQETVECLRKFNARRKLKGAILTTMLVSRNFS**ACKSLLNKKSDGVKKRKSSSSVYLMGSTESCNTTEEEDMKGRK**ARKQEIIKITEQLIEAINNGDFEAYTRICDPGLTSFEPEALGNLVEGMDFHKFYFENLLSKNSKPVHTTILNPHVHLIGE

**S4 Table. Alternative *camk2* splice variants identified in wild type kidney marrow lymphocytes.**
