# Supplementary figures and images for: Constitutively active CaMKII Drives B lineage acute lymphoblastic leukemia/lymphoma in tp53 mutant zebrafish
Source: PLoS Genet. 2023 Dec 20;19(12):e1011102. doi: 10.1371/journal.pgen.1011102 (PMC10766190; doi:10.1371/journal.pgen.1011102)

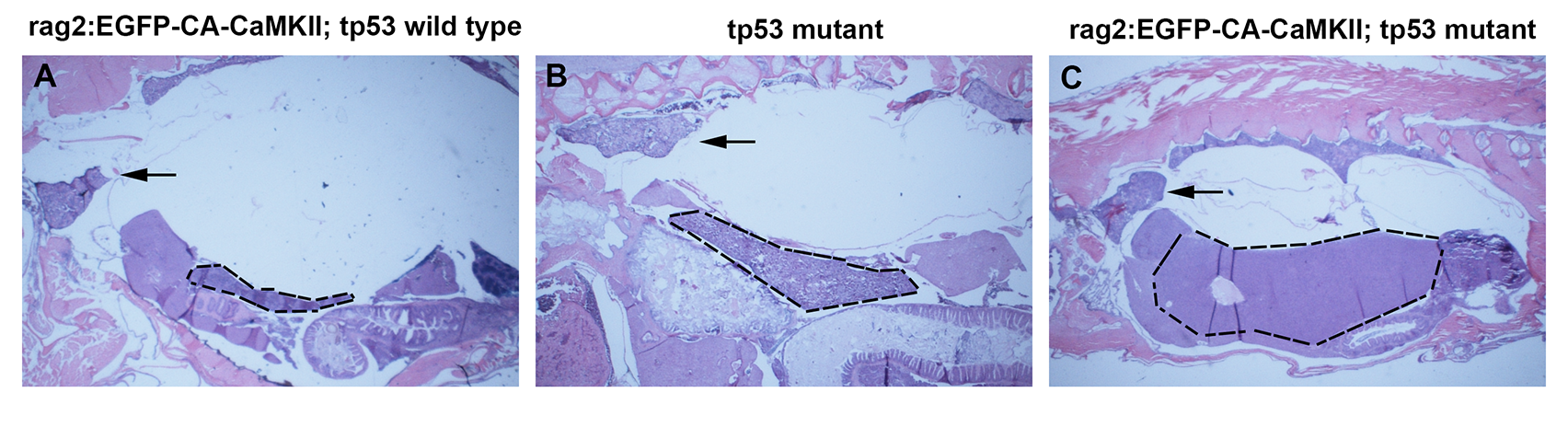

Supplement: S1 Fig — (A,B) Spleen (outlined by dashed lines) size in rag2:EGFP-CA-CaMKII transgenic and tp53 mutant animals is normal compared to the enlarged spleen seen in (C) rag2:EGFP-CA-CaMKII; tp53 mutants. Head kidney (arrow) is also pictured. (TIF) [file pgen.1011102.s006.tif]

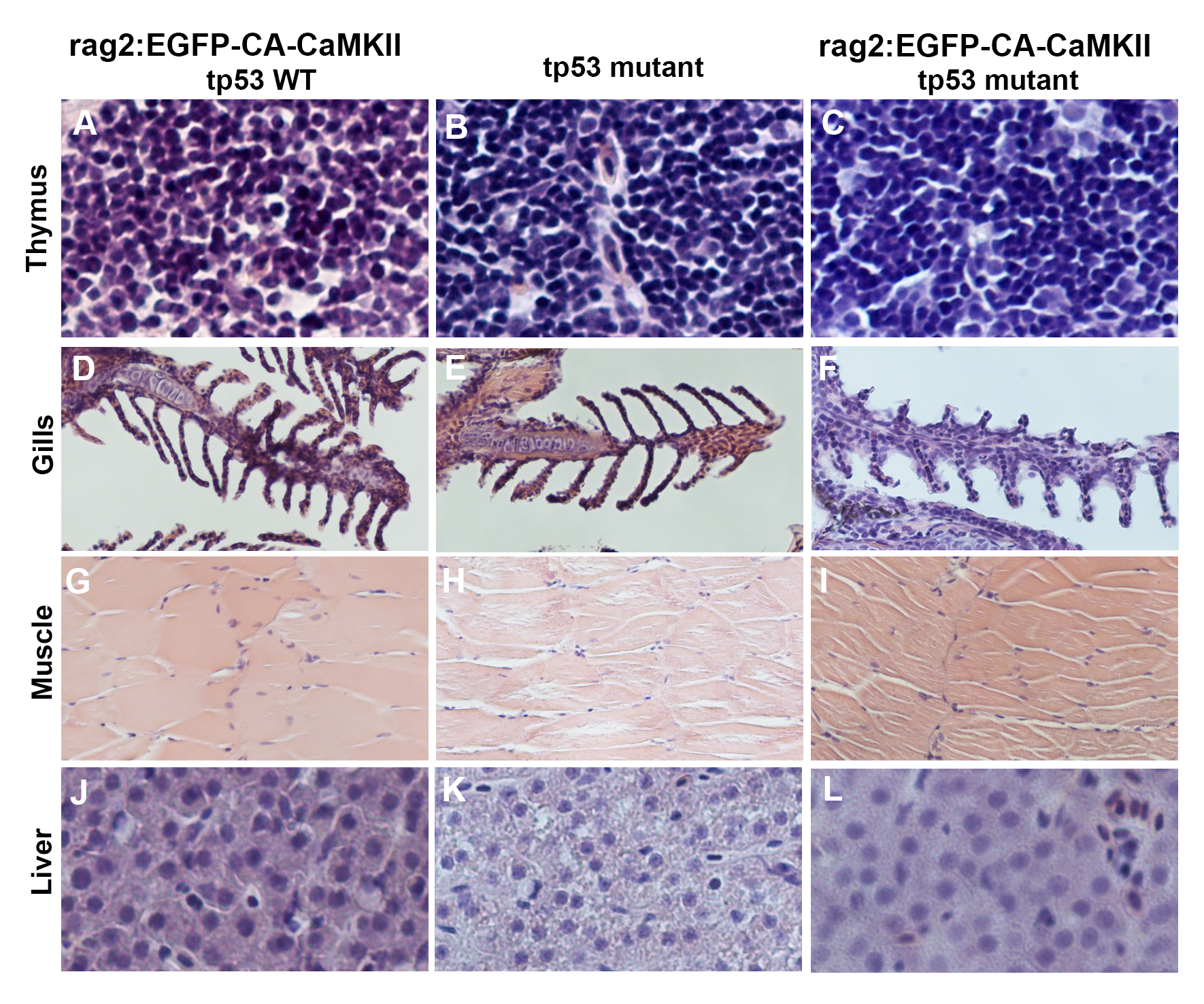

Supplement: S2 Fig — Histological sections are normal in rag2:EGFP-CA-CaMKII wild type, tp53 mutant, and rag2:EGFP-CA-CaMKII; tp53 mutant fish for (A-C) thymus, (D-F) gills, (G-I) muscle, and (J-L) liver. (TIF) [file pgen.1011102.s007.tif]
